# Spontaneous formation of MXene-oxidized sono/chemo-dynamic sonosensitizer/nanocatalyst for antibacteria and bone-tissue regeneration

**DOI:** 10.1186/s12951-023-01933-z

**Published:** 2023-06-14

**Authors:** Yang Yu, Houyi Sun, Qunshan Lu, Junyuan Sun, Pengfei Zhang, Linran Zeng, Krasimir Vasilev, Yunpeng Zhao, Yu Chen, Peilai Liu

**Affiliations:** 1 Department of Orthopaedics, Qilu Hospital of Shandong University, No. 107 West Wenhua Road, Jinan, Shandong 250012 People’s Republic of China; 2 Materdicine Lab, School of Life Sciences, Shanghai University, Shanghai, 200444 People’s Republic of China; 3 Laboratory of Basic Medical Sciences, Qilu Hospital of Shandong University, Jinan, 250012 People’s Republic of China; 4 The 1st Affiliated Hospital of Kunming Medical University, Kunming Yunnan, 650032 People’s Republic of China; 5 College of Medicine and Public Health, Flinders University, Sturt Road, Bedford Park, SA 5042 Australia; 6 Academic Unit of STEM, University of South Australia, Mawson Lakes, Adelaide, SA 5095 Australia

**Keywords:** Prosthetic joint infections, Nanosheets, Sonodynamic therapy, Synergistic antibacterial activity, Bone regeneration

## Abstract

Prolonged and incurable bacterial infections in soft tissue and bone are currently causing large challenges in the clinic. Two-dimensional (2D) materials have been designed to address these issues, but materials with satisfying therapeutic effects are still needed. Herein, CaO_2_-loaded 2D titanium carbide nanosheets (CaO_2_-TiO_x_@Ti_3_C_2_, C-T@Ti_3_C_2_) were developed. Surprisingly, this nanosheet exhibited sonodynamic ability, in which CaO_2_ caused the in situ oxidation of Ti_3_C_2_ MXene to produce acoustic sensitiser TiO_2_ on its surface. In addition, this nanosheet displayed chemodynamic features, which promoted a Fenton reaction triggered by self-supplied H_2_O_2_. We detected that C-T@Ti_3_C_2_ nanosheets increased reactive oxygen species (ROS) production in response to sonodynamic therapy, which displayed an ideal antibacterial effect. Furthermore, these nanoreactors facilitated the deposition of Ca^2+^, which promoted osteogenic transformation and enhanced bone quality in osteomyelitis models. Herein, a wound healing model and prosthetic joint infection (PJI) model were established, and the C-T@Ti_3_C_2_ nanosheets played a protective role in these models. Taken together, the results indicated that the C-T@Ti_3_C_2_ nanosheets function as a multifunctional instrument with sonodynamic features, which might reveal information regarding the treatment of bacterial infections during wound healing.

**Graphical Abstract:**

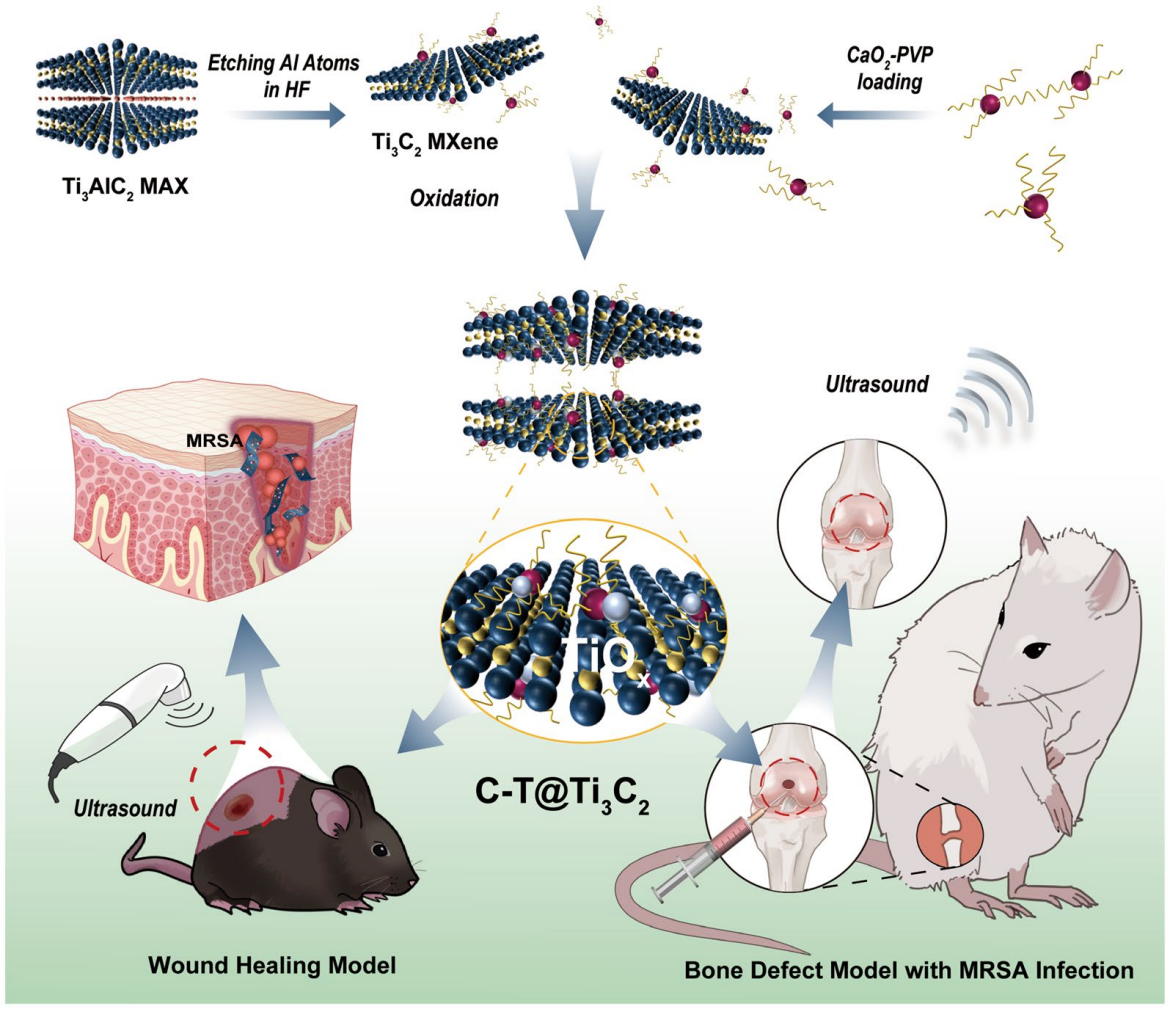

**Supplementary Information:**

The online version contains supplementary material available at 10.1186/s12951-023-01933-z.

## Background

Although prosthetic joint replacements provide excellent functional improvements, improve the quality of life of patients and offer pain relief [1], approximately 2% of patients who undergo surgery experience complications by prosthetic joint infections (PJIs). It is estimated that the incidence of PJIs in the United States might increase to 6.5% by 2030 [1, 2]. Infections caused mainly by *Staphylococcus aureus* (*S. aureus*) slow the healing process and lead to bone loss [3–5]. The extensive surgical interventions and emergence of multidrug resistant (MDR) bacteria have substantially increased the suffering and financial burden on patients [6–9]. Therefore, it is necessary to explore multifunctional biomaterials with antibacterial activity while promoting bone regeneration for orthopaedic surgical treatment.

Recent research has indicated that MXene, a new two-dimensional (2D) nanomaterial with ultrathin layer structure topology, has been widely used in the biomedical field. As transition metal carbides, MXenes feature excellent metallic conductivity, photothermal-conversion capability, hydrophilicity and mechanical properties [10, 11] as well as a high specific surface area; as a result, MXenes have application in nanomedicine fields, such as phototherapy, drug delivery, biomedical imaging, biosensing and even tissue engineering [12–19]. Photothermal therapy (PTT) has been extensively investigated for treating tumours due to the excellent photothermal conversion efficiency of MXenes [20–23]. However, the limited penetration of light into deep tissues of PTT makes the treatment much less effective, which limits its use in treating deep-seated diseases [16, 24, 25]. To date, sonodynamic therapy (SDT), which combines ultrasound (US) with acoustic sensitizers, has shown high therapeutic potential in antitumour, antibacterial and bone repair treatments due to its desirable ability to penetrate tissues and high biosafety to the human body [15, 26–28]. Chemodynamic therapy (CDT), another reactive oxygen species (ROS)-based therapeutic modality, can convert H_2_O_2,_ which is less efficient in treating infection, into ROS with higher cytotoxicity by catalysing metal ion-mediated Fenton or Fenton-like reactions in acidic microenvironments [16, 29–33]. It has been reported that CDT and SDT display synergistic effects, which markedly enhance the catalytic efficiency in infection [34]. In addition, increased the O_2_ secreted through this synergistic therapeutic strategy inhibits the growth of anaerobic bacteria [34–37]. It is known that cells in organisms are in a slightly alkaline environment, and the amount of basal ROS in normal cells is relatively low; therefore, the ROS production of SDT under moderate US induction can kill bacteria while the normal cells remain safe [38–40].

Herein, we report a self-supplied H_2_O_2_-triggered and SDT-strengthened Fenton reaction based on the constructed 2D therapeutic CaO_2_-TiO_x_@Ti_3_C_2_ (designated C-T@Ti_3_C_2_) nanomedicine with biosafety and multifunctions, including antibacterial effects and enhancement of bone repair. The C-T@Ti_3_C_2_ nanoworks employ calcium peroxide (CaO_2_) as an efficient source of H_2_O_2_ to sustain the Ti_3_C_2_ MXene nanosheet-mediated Fenton catalytic reaction, releasing more toxic ·OH to induce bacterial death. Intriguingly, by utilizing CaO_2_ oxidation, the in situ oxidation of Ti_3_C_2_ MXene generates sonosensitizer TiO_2_ on the MXene surface, accompanied by the production of a small amount of trivalent titanium ions (Ti^3+^) [41, 42], which can further activate CDT by catalysing the formation of ·OH and O_2_ from H_2_O_2_ through a Fenton-like reaction [25, 42]. Through US excitation, TiO_2_ can increase ROS (·OH, ·O_2_) production and subsequently mediate bacterial apoptosis. Additionally, MXenes can effectively trap photogenerated holes to compensate for the defects in TiO_2_ with a fast electron (e^−^)-hole (H^+^) complexation rate (50 ± 30 ns) [43]; thus, MXenes effectively enhance the catalytic activity of TiO_x_ (TiO_2_ and Ti^3+^). The regeneration process of bone defects includes antibacterial reactions, which might otherwise cause delayed healing or even osteomyelitis, and the deposition of calcium ions in the infected bone defect, providing the raw material for bone repair [44, 45]. Overall, we demonstrated the physicochemical properties of C-T@Ti_3_C_2_ in terms of morphology, particle size and lattice structure and the combined effects of C-T@Ti_3_C_2_ in the direction of bacterial inhibition and osteogenesis in vivo and in vitro.

## Results and discussion

### Synthesis and characterization of the 2D C-T@Ti_3_C_2_ nanosheets

**Scheme 1.**
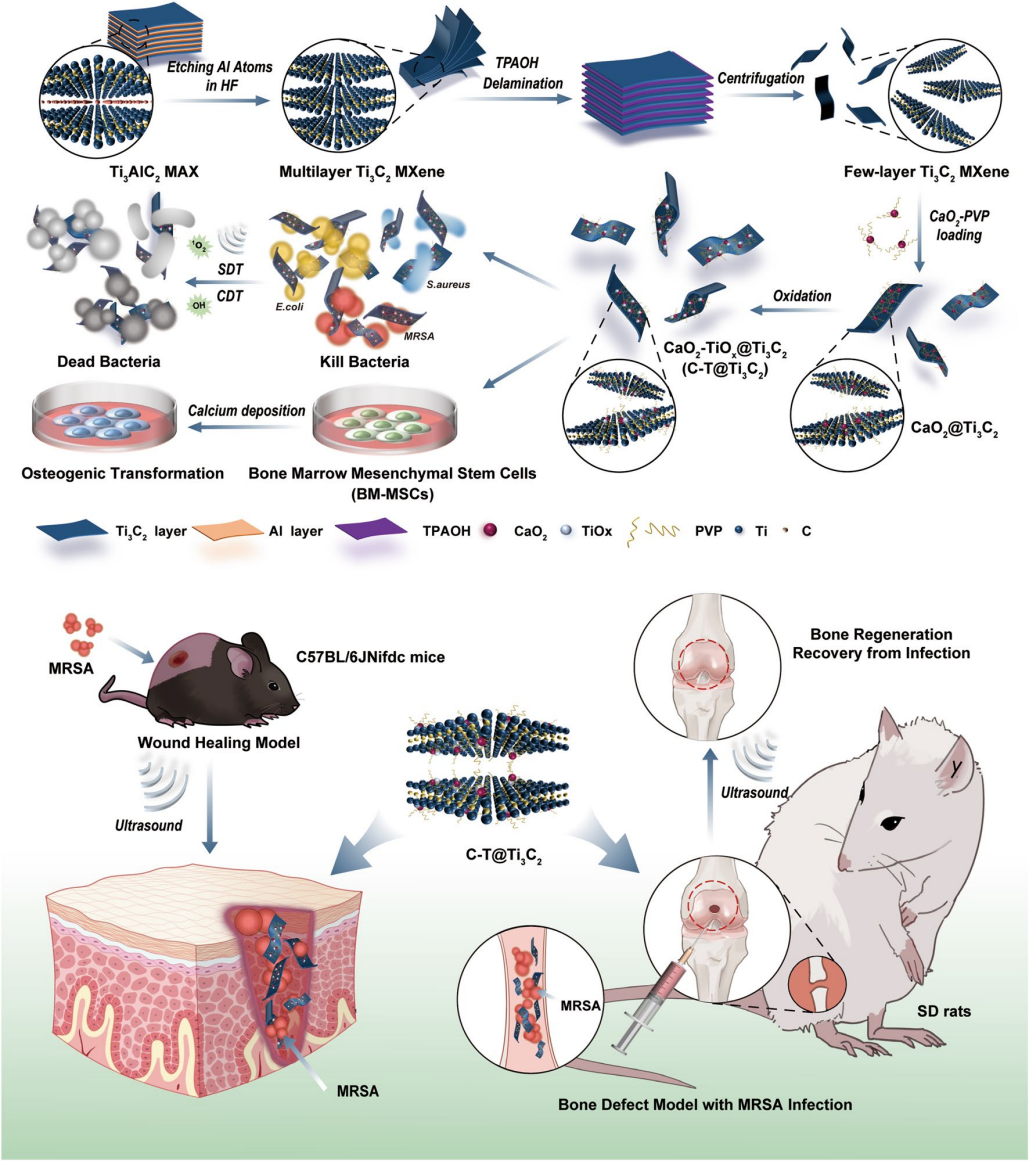
Schematic illustration of the synthesis and performance of C-T@Ti_3_C_2_ nanosheets. The synthesis of C-T@Ti_3_C_2_ nanosheets and their in vitro as well as in vivo application in bacterial infections, especially in deep infections induced by MDR bacteria, and the repair of bone defects

**Fig. 1:**
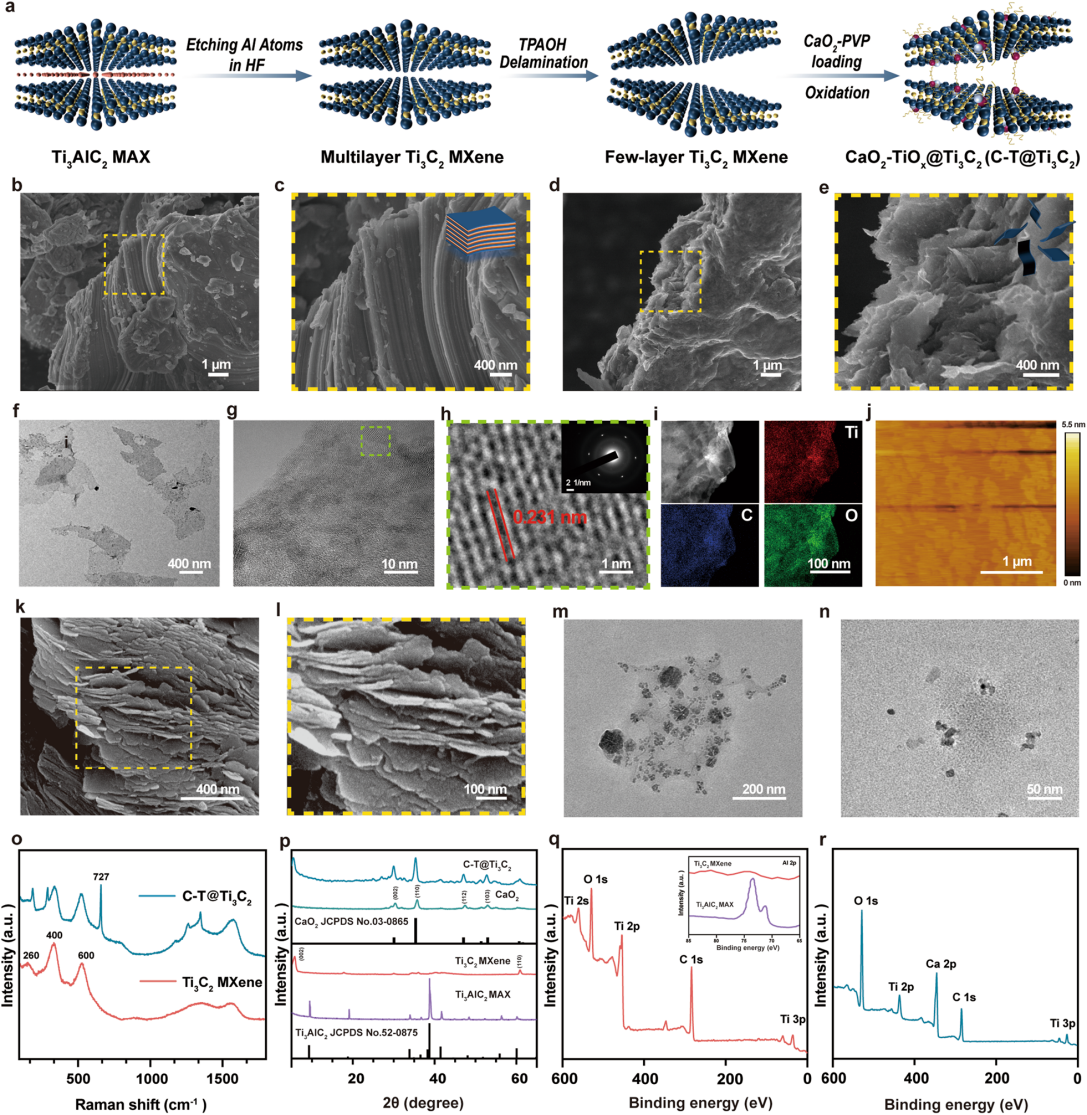
Characterization of Ti_3_C_2_ MXene and C-T@Ti_3_C_2_ nanosheets. **a** Schematic diagram for the fabrication of Ti_3_C_2_ MXene and C-T@Ti_3_C_2_ nanosheets. **b**, **c** SEM images of Ti_3_AlC_2_ MAX powder presenting a dense lamellar structure containing impacted layers. **d**, **e** SEM, **f** TEM, **g**, **h** HRTEM, **i** dark-field STEM images and corresponding element mappings (for Ti, C, and O) and **j** AFM image of FL Ti_3_C_2_ MXene after intercalation with TPAOH. **k**, **l** SEM and **m** TEM images of C-T@Ti_3_C_2_ nanosheets. **n** TEM images of CaO_2_ NPs. **o** Raman spectra of Ti_3_C_2_ MXene and C-T@Ti_3_C_2_ nanosheets. **p** XRD patterns of Ti_3_AlC_2_ MAX powder, Ti_3_C_2_ MXene, CaO_2_ NPs and C-T@Ti_3_C_2_ nanosheets. **q**, **r** XPS curves for (**q**) Ti_3_C_2_ MXene and (**r**) C-T@Ti_3_C_2_ nanosheets. Representative images of three replicates of each group are shown

The 2D Ti_3_C_2_ MXene was synthesized by a facile exfoliation and intercalation process [19, 25]. The aluminium (Al) layers were selectively removed from the corresponding MAX phase precursor Ti_3_AlC_2_ by chemical etching using a hydrofluoric acid (HF) solution. The layers were then further stripped by intercalation of tetrapropylammonium hydroxide (TPAOH) to reduce interlayer interactions, followed by centrifugation to obtain few-layer (FL) Ti_3_C_2_ MXene (Scheme 1 and Fig. 1a). The scanning electron microscope (SEM) image demonstrates that the pristine Ti_3_AlC_2_ exhibits a dense compacted laminar plate-like structure (Fig. 1b, c). After etching, the dense layered structure transforms into a loose accordion multilayer (ML) structure (Additional file 1: Fig. S2) and the FL configurations (Fig. 1d, e). The TEM images of Ti_3_C_2_ MXene nanosheets show a sheet-like morphology (Fig. 1f). The prepared Ti_3_C_2_ MXene is highly hydrophilic and features a large number of hydrophilic oxygen-containing groups on its surface, such as –OH and –COOH; these are functional groups that enable strong connections to be formed between Ti_3_C_2_ MXene and numerous functional nanoparticles (NPs) [46]. Figure 1g and h shows a typical high-resolution transmission electron microscope (HRTEM) micrograph of Ti_3_C_2_ MXene, and the corresponding original selected area electron diffraction (SAED) in the upper right corner confirms its hexagonal symmetrical structure. The lattice spacing was calculated to be 0.231 nm [47]. In addition, the corresponding elemental surface distribution clearly shows that the Ti, C and O elements were uniformly distributed on the Ti_3_C_2_ MXene nanosheets (Fig. 1i). Atomic force microscopy (AFM) images confirmed the formation of FL Ti_3_C_2_ MXene, and the thickness of the Ti_3_C_2_ nanosheets was approximately 2–3 nm (Fig. 1j). Furthermore, CaO_2_ NPs were originally synthesized by reacting Ca(OH)_2_ with H_2_O_2_ under alkaline conditions. TEM (Fig. 1n) and dynamic light scattering (DLS) measurements show that the average hydrodynamic size of CaO_2_ NPs is approximately 10 nm (Additional file 1: Fig. S4). The CaO_2_ NPs were efficiently loaded onto the surface of ammoniated Ti_3_C_2_ MXene (Ti_3_C_2_-NH_2_) by vigorous stirring to obtain C-T@Ti_3_C_2_ nanosheets. Figure 1k, l and m are SEM and TEM images of C-T@Ti_3_C_2_, respectively. The typical morphology of the nanosheets and the uniform distribution of CaO_2_ NPs on the surface of the nanosheets were observed (Scheme 1, Fig. 1a). Moreover, the corresponding elemental surface distribution clearly shows that the Ti, Ca, C and O elements are evenly distributed on the C-T@Ti_3_C_2_ nanosheets (Additional file 1: Fig. S3). Digital photographs show their excellent dispersibility and hydrophilicity (Additional file 1: Fig. S1).

We tested the Raman spectra of Ti_3_C_2_ MXene and C-T@Ti_3_C_2_ nanosheets to illustrate the surface oxidation in their nanostructures (Fig. 1o). There are three distinctive peaks of Ti_3_C_2_ MXene that are located at 254, 413 and 610 cm^−1^ [48, 49]. However, these peaks appear blue-shifted in the Raman spectrum of C-T@Ti_3_C_2,_ and a sharp characteristic peak appears at 727 cm^−1^. Calcium peroxide (CaO_2_) is an efficient source of H_2_O_2_, which sustains the Fenton-catalysed reaction mediated by Ti_3_C_2_ MXene nanosheets. The intensities of the D and G peaks in the C-T@Ti_3_C_2_ Raman spectra are significantly increased, indicating the carbon content in the C-T@Ti_3_C_2_ nanosheets is enhanced. Moreover, a sharp characteristic peak appears at 727 cm^−1^ in the Raman spectra of the C-T@Ti_3_C_2_ nanosheets. The result fully demonstrates the combination of TiO_x_ and CaO_2_ generated on Ti_3_C_2_ MXene.

We further used X-ray diffraction (XRD) to analyse the structure of TiAlC_2_ MAX, Ti_3_C_2_ MXene nanosheets and C-T@Ti_3_C_2_ nanosheets (Fig. 1p). Compared with the XRD diffraction patterns of pristine Ti_3_AlC_2_ MAX, the Ti_3_C_2_ MXene nanosheets showed an intense peak (002) at 2θ ≈ 6°. The most prominent peak at 2θ ≈ 39° disappears for the etched pristine Ti_3_AlC_2_ MAX by HF etching and TPAOH intercalation, which further indicates that the Al atoms in the pristine Ti_3_AlC_2_ MAX have been etched away effectively. Additionally, 2θ values of 30.1°, 35.6°, 47.3° and 52.8° correspond to the (002), (110), (112) and (103) lattice planes of CaO_2_ NPs in the XRD pattern of C-T@Ti_3_C_2_ nanosheets, which illustrates that CaO_2_ NPs are present on the surface of Ti_3_C_2_ nanosheets. This characterization also further demonstrates that the Ti_3_C_2_ MXene nanosheets were successfully constructed and CaO_2_ NPs immobilized onto their surface.

**Fig. 2:**
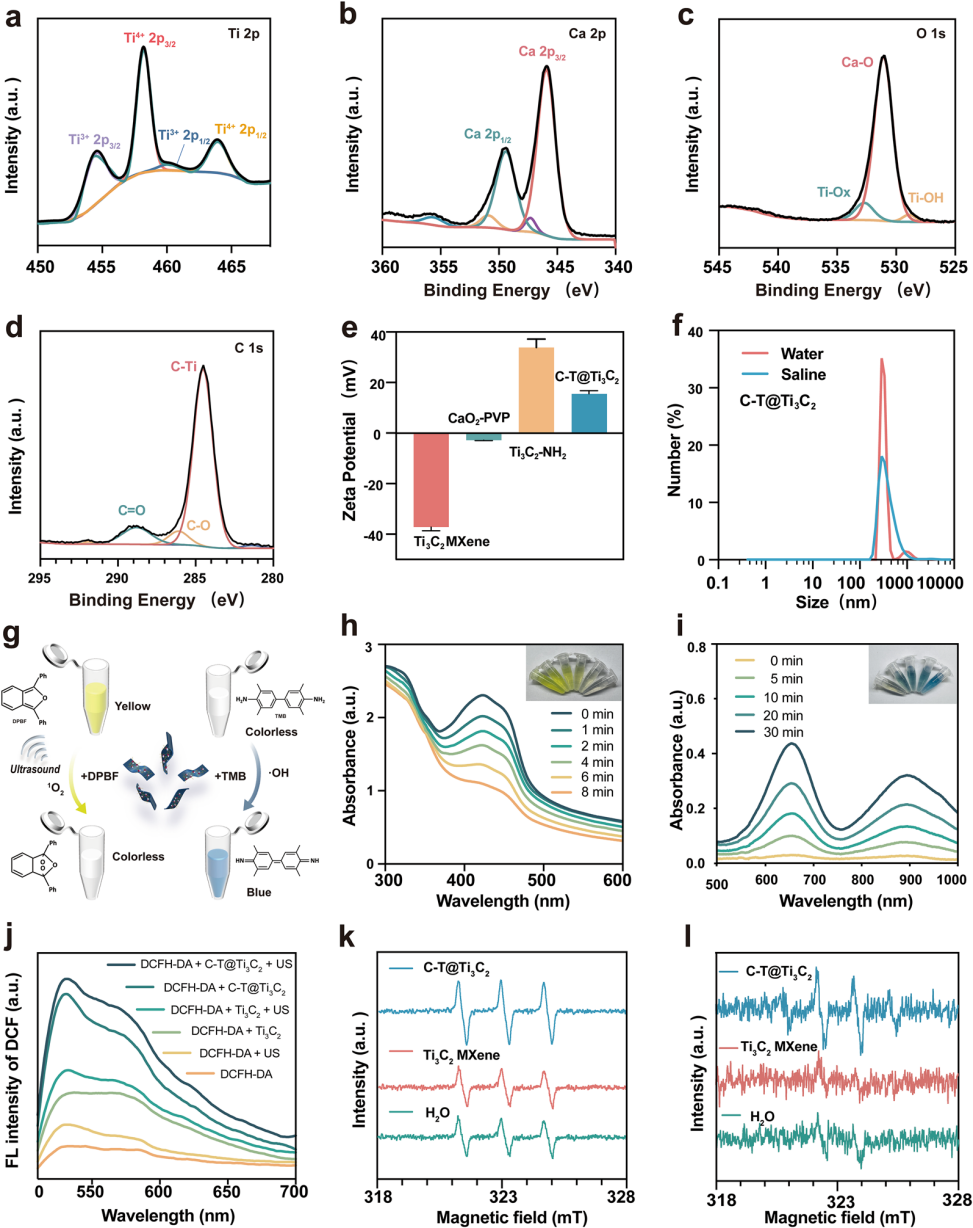
Sono/chemodynamic performances of the C-T@Ti_3_C_2_ nanosheets. **a**–**d** XPS spectra of (**a**) Ti 2p, (**b**) Ca 2p, (**c**) O 1 s and (**d**) C 1 s in C-T@Ti_3_C_2_ nanosheets. **e** Zeta potentials of Ti_3_C_2_ MXene nanosheets, CaO_2_ NPs, Ti_3_C_2_-NH_2_, and C-T@Ti_3_C_2_ composite nanosheets. **f** Particle-size distribution of the C-T@Ti_3_C_2_ nanosheets dispersed in water and saline as tested by DLS. **g** Schematic illustration of the SDT and CDT performances of C-T@Ti_3_C_2_ nanosheets via the DPBF probe and the TMB probe, respectively. **h**, **i** Comparison of (**h**) SDT performances of C-T@Ti_3_C_2_ nanosheets via the amount of DPBF degradation by over different oxidation durations and (**i**) CDT performances of C-T@Ti_3_C_2_ nanosheets using TMB probe over different oxidation durations. **j** Fluorescence changes in DCFH-DA in the presence of different materials upon US irradiation. **k**, **l** ESR spectra of (**k**) singlet oxygen and (**l**) hydroxyl radical detection using TEMP and DMPO, respectively, as spin trapping agents after different treatments

To further clarify the changes in the products during the reaction stage and the surface composition of the final C-T@Ti_3_C_2_ nanosheets, X-ray photoelectron spectroscopy (XPS) characterization of Ti_3_C_2_ MXene and C-T@Ti_3_C_2_ nanosheets was performed to obtain more detailed information. In the full spectra of Ti_3_C_2_ MXene and C-T@Ti_3_C_2_ nanonetworks (Fig. 1q, r), the peak intensity of Ti_3_C_2_ MXene at the Al element decreases significantly relative to Ti_3_AlC_2_ MAX, which confirms that the Al component was efficiently etched after HF etching. In addition, the analysis of the corresponding Ca, C and O elements (Fig. 2a–d), both Ti^4+^ and Ti^3+^ were found in the synthesized C-T@Ti_3_C_2_ nanosheets. The binding energy peaks at 464.5 and 458.7 eV can be attributed to Ti^4+^ 2p_1/2_ and Ti^4+^ 2p_3/2_, while the smaller peaks at 463.5 and 457.9 eV correspond to the electrons of Ti^3+^ 2p_1/2_ and Ti^3+^ 2p_3/2_, respectively [37]. This indicates that a TiO_x_ component containing both Ti^3+^ and Ti^4+^ was obtained, which provides support that C-T@Ti_3_C_2_ exhibits potential SDT and CDT abilities.

The variation in zeta potential between Ti_3_C_2_ MXene, CaO_2_ NPs, Ti_3_C_2_-NH_2_ and C-T@Ti_3_C_2_ is shown in Fig. 2e. The initial Ti_3_C_2_ MXene nanosheets showed a negative zeta potential (− 37.2 mV) due to the hydroxyl groups on the surface, so amine groups were introduced using (3-aminopropyl)triethoxysilane (APTES). Hydrolysis reaction between silanol and hydroxyl groups on MXene covalently anchored amino groups on the surface of nanosheets. The groups produced the negative Zeta potential positive (+ 33.8 mV) (pH = 6.0), which facilitated subsequent loading with the negatively charged CaO_2_ NPs. To improve the stability of the nanosheets in the physiological environment, the surface of the C-T@Ti_3_C_2_ nanosheet was modified with polyvinylpyrrolidone (PVP) (Fig. 2f). The C-T@Ti_3_C_2_ nanosheets modified with PVP were stable in both aqueous and salt solutions and varied less, and the particle size of the nanosheets was approximately 300–400 nm, whereas the stability of the unmodified C-T@Ti_3_C_2_ varied considerably in aqueous and physiological environments, as determined by DLS (Additional file 1: Fig. S5).

### Sonodynamic and chemodynamic performance of the C-T@Ti_3_C_2_ nanosheets

To further validate the ability of the C-T@Ti_3_C_2_ nanosheets to generate ROS, as well as their CDT and SDT abilities, an ROS production assay was performed by corresponding chemical probes (Fig. 2g–i). The sonodynamic properties of the C-T@Ti_3_C_2_ nanosheets were evaluated using the typical 1,3-diphenylisobenzofuran (DPBF) as a molecular probe for ROS (^1^O_2_) detection. The intensity of the characteristic absorption peak of DPBF at 416 nm decreased markedly in the presence of C-T@Ti_3_C_2_ nanosheets (Fig. 2h) under US stimulation (1 Wcm^−2^, 50% duty cycle, 1 MHz), while no significant difference was observed in the absorption spectra without the addition of C-T@Ti_3_C_2_ nanosheets (Additional file 1: Fig. S6). This result suggested that the C-T@Ti_3_C_2_ nanosheets exhibited the acoustic kinetic ability to induce ROS production for the degradation of DPBF. In contrast, to verify the CDT effect of C-T@Ti_3_C_2_ nanosheets, the typical 3,3,5,5-tetramethylbenzidine (TMB) was used as a probe for ·OH without US activation. The reaction mechanism of H_2_O_2_-mediated oxidation of TMB could be divided into two steps. The O–O bond in the H_2_O_2_ molecule was broken to form ·OH, and TMB was subsequently oxidized by ·OH to form ox TMB. After different reaction times, C-T@Ti_3_C_2_ nanosheets showed the ability to induce a Fenton-like reaction to produce ·OH.

The ability of C-T@Ti_3_C_2_ nanosheets to generate ROS in vitro was measured using nonfluorescent 2ʹ,7ʹ-dichlorofluorescein diacetate (DCFH-DA), which can be oxidized by ROS to generate fluorescent 2',7'-dichlorofluorescein (DCF). As shown in Fig. 2j, the fluorescence intensity of C-T@Ti_3_C_2_ + US (1 Wcm^−2^, 50% duty cycle, 1 MHz, 5 min) was stronger than that of Pd@Pt-T790, indicating that CDT combined with US-triggered SDT produced more ROS than that of CDT alone. Ti_3_C_2_ MXene produced less ROS with ultrasound or without ultrasound treatment, while DCFH-DA alone or DCFH-DA treated with US showed almost no fluorescence, indicating negligible ROS production.

Electron spin resonance (ESR) spectroscopy was used to evaluate the production of ^1^O_2_ and ·OH by using the electron trapping agents 2,2,6,6-tetramethylpiperidine (TEMP) and dimethyl pyridine N-oxide (DMPO). The ^1^O_2_ peak intensity of the C-T@Ti_3_C_2_ + US group was significantly more robust than that of the US and Ti_3_C_2_ MXene groups (Fig. 2k), indicating that US irradiation effectively induced ^1^O_2_ production assisted by C-T@Ti_3_C_2_ nanosheets. Similarly, it produced higher ·OH content than that of the other groups (Fig. 2l). Combined with the XPS characterization, Ti^3+^ and Ti^4+^ were produced by oxidation in C-T@Ti_3_C_2_, and the interconversion between the two forms enhanced the effects of SDT and CDT [41]. Without US irradiation, Ti^3+^ triggered a Fenton-like reaction and produced ·OH. SDT took effect after US stimulation, and the US activated TiO_x_ to produce free e^−^, which rapidly combined with adjacent O_2_ to produce ^1^O_2_, while h^+^ was transferred to the hydroxyl-capped Ti_3_C_2_ MXene surface [50].

### In vitro antibacterial ability based on the C-T@Ti_3_C_2_ nanosheets

**Fig. 3:**
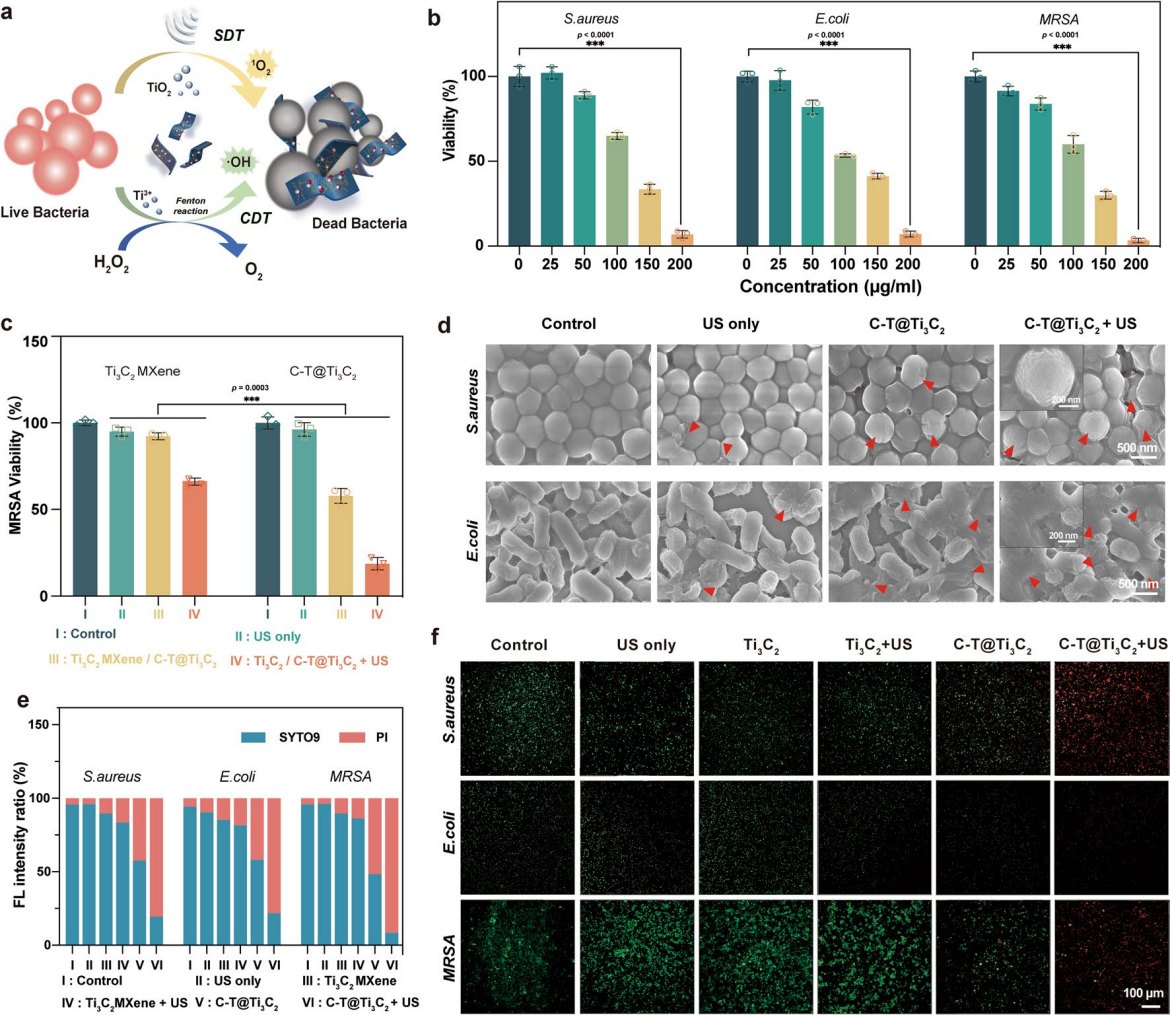
In vitro antibacterial evaluation of C-T@Ti_3_C_2_ nanosheets. **a** Schematic diagram of the mechanism of bactericidal action of C-T@Ti_3_C_2_-mediated SDT&CDT. **b** Bacterial viability of *E. coli*, *S. aureus* and *MRSA* colonies after treatment with different concentrations of C-T@Ti_3_C_2_ nanosheets (n = 3 for each group). **c** Viability of *MRSA* after different treatments (n = 3 for each group). **d** SEM images of the microscopic morphology of bacteria obtained for *E. coli* and *S. aureus* after different treatments. **e** Corresponding fluorescence intensity analysis for SYTO9/PI costaining from (**f**). **f** Fluorescence microscopy images of *MRSA* stained by SYTO-9 and propidium iodide (PI) after various treatments (green fluorescence, SYTO-9 representing living cells; red fluorescence, PI representing dead cells). Data are presented as the mean ± SD, and statistical significance was calculated using the two-tailed t test and two-way analysis of variance (ANOVA) test, ^*^P < 0.05, ^**^P < 0.01, ^***^P < 0.001. One representative image of three replicates from each group is shown

To evaluate the antibacterial properties of C-T@Ti_3_C_2_ nanosheets, Escherichia coli (*E. coli*, gram-negative bacterium), Staphylococcus aureus (*S. aureus*, gram-positive bacterium) and methicillin-resistant Staphylococcus aureus (*MRSA*, gram-positive bacterium) were treated with C-T@Ti_3_C_2_ nanosheets (Fig. 3a). As shown in Fig. 3b, we coincubated different concentrations of C-T@Ti_3_C_2_ with three bacteria. In the absence of US, C-T@Ti_3_C_2_ with a concentration greater than 150 µg/mL could reduce bacterial viability by more than 50%. At a concentration of 200 µg/mL, the activity of the bacteria was markedly decreased. Next, the antibacterial property of the C-T@Ti_3_C_2_ nanosheets was evaluated in vitro under US stimulation (1 Wcm^−2^, 50% cycle, 1 MHz, 5 min), and we compared the antibacterial property of Ti_3_C_2_ and C-T@Ti_3_C_2_ nanosheets (Fig. 3c and Additional file 1: Fig. S7). The results showed that the C-T@Ti_3_C_2_ nanosheets showed an advantage over the Ti_3_C_2_ MXene at the same dose (100 μg mL^−1^).

Moreover, the effect of C-T@Ti_3_C_2_ nanosheets on the morphology of bacteria was observed by SEM (Fig. 3d). Incubation with C-T@Ti_3_C_2_ nanosheets for 1 h resulted in irreversible damage to the bacterial membrane. By combining C-T@Ti_3_C_2_ with US stimulation, the bacteria exhibited a contracted, ruptured or even completely lysed morphology. In contrast, SEM images of *E. coli* and *S. aureus* in the other treatments showed that the morphology of the bacteria remained largely unchanged or slight distortions or slight wrinkles could be observed.

To further assess the antibacterial performance of the different samples, live/dead costaining with SYTO9 (green, live bacteria) and propidium iodide (PI, red, dead bacteria) was performed and analysed regarding the signal intensity and ratio (Fig. 3e, f). As shown in Fig. 3f, there was no significant difference between the control, US and Ti_3_C_2_ MXene groups, but the combination of US resulted in an increased proportion of dead bacteria, which might be associated with the activation of the SDT effect by Ti_3_C_2_ MXene in the presence of US. In the absence of US, no bactericidal activity was observed for Ti_3_C_2_ MXene, while C-T@Ti_3_C_2_ exhibited a weaker bactericidal effect. C-T@Ti_3_C_2_ nanosheets could still produce O_2_ in the absence of US, so the proportion of bacteria was also reduced compared with that of the control group in the stained pictures. The C-T@Ti_3_C_2_ + US group showed very few live bacteria in comparison, indicating its strong antibacterial ability. The ratio of red fluorescence intensity was 80.6%, 78.3% and 91.6%, respectively, to the overall signal, which was much higher than the other groups of treated bacteria due to the synergistic effect of its SDT and CDT.

Since the production of ROS is a signal of cellular redox homeostasis [51], to explore the bactericidal mechanism of C-T@Ti_3_C_2_-induced SDT, DCFH-DA was used to detect ROS in *MRSA* (Additional file 1: Fig. S8). Fluorescence images of bacteria treated with C-T@Ti_3_C_2_ + US showed strong green fluorescence, indicating that a significant amount of ROS was produced by SDT mediated by the C-T@Ti_3_C_2_ nanosheets. *MRSA* treated with C-T@Ti_3_C_2_ alone also produced some ROS. In addition, bacteria treated with Ti_3_C_2_ MXene + US also showed faint green fluorescence, as Ti_3_C_2_ MXene could also activate SDT, while the other three groups produced virtually no ROS.

### Bactericidal mechanism of the C-T@Ti_3_C_2_ nanosheets

**Fig. 4:**
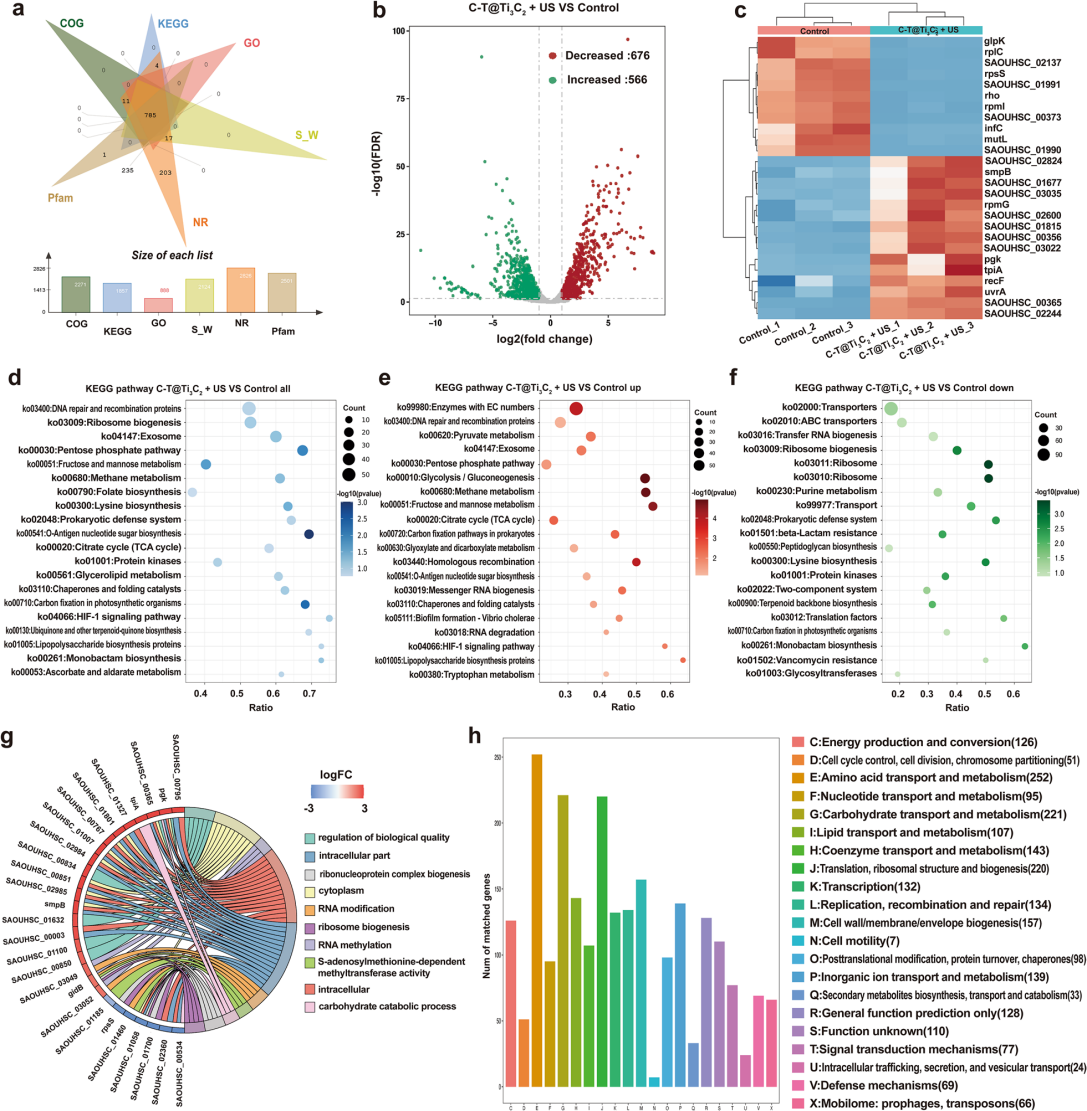
RNA sequence analysis of the bactericidal mechanism of C-T@Ti_3_C_2_ nanosheet-mediated SDT. **a** Venn diagram showing the genes in the control and C-T@Ti_3_C_2_ + US groups. **b** Volcano map of differential gene expression. **c** Heatmaps of the hierarchical cluster analysis of the screened differentially expressed genes and the cluster genes with the same or similar expression behaviour. **d**–**f** Bubble diagram for KEGG enrichment analysis of differentially expressed genes, (**d**) all, (**e**) down- and (**f**) upregulated genes. **g** Chord diagram of the sequences of highly expressed genes analysed from a highly regulated GO pathway. h Gene COG classification statistics chart

RNA sequencing analysis was then used to illuminate the differences in gene expression of *MRSA* after treatment with C-T@Ti_3_C_2_ nanosheets under US stimulation to clarify the bactericidal mechanism of C-T@Ti_3_C_2_ nanosheets via CDT and SDT. A collection of similar genes recorded in six databases (COG, KEGG, GO, S_W, NR and Pfam) is plotted in Fig. 4a, in which a total of 785 genes were annotated. As shown in Fig. 4b, there were 566 and 676 differentially expressed gene sequences, which were upregulated and downregulated in the C-T@Ti_3_C_2_ + US group, respectively, compared to the control group. A heatmap was used to analyse the difference in gene expression between the control and C-T@Ti_3_C_2_ + US groups (Fig. 4c). In the heatmap, the expression levels of the gpk and tpiA genes were upregulated, which were related to carbohydrate metabolism. In addition, the expression of genes (SAOUHSC_02137, SAOUHSC_01990 and SAOUHSC_01991) associated with cell membrane transport proteins was decreased. Importantly, the expression of genes (rpsS, inf-3, mutL, rplC and rpmG) associated with protein synthesis was also reduced. This may be related to the inhibition of ribosome function by C-T@Ti_3_C_2_. Increased expression of recF and uvrA genes is associated with DNA replication [52, 53]. The expression of recF and uvrA genes, which are related to DNA replication, was also elevated.

As shown in Fig. 4d–f, based on KEGG enrichment analysis, pathways such as the pentose phosphate pathway, methane metabolism, citrate cycle (TCA cycle) and O-antigen nucleotide sugar biosynthesis are clearly associated with increased gene expression in response to C-T@Ti_3_C_2_ + US stimulation, while the genes in lysine biosynthesis, prokaryotic defence system, ribosome, ribosome biogenesis, protein kinases and monobactam biosynthesis were decreased. In addition, the expression levels of genes belonging to intracellular structure, regulation of biological quality, RNA methylation and cytoplasm, such as pgk, tpla, and gene (SAOUHSC_0083), were downregulated. In contrast, the expression levels of rpsS and the gene (SAOUHSC_01185), etc., regarding ribonucleoprotein complex biogenesis, ribosome biogenesis, and S-adenosylmethionine-dependent methyltransferase activity, respectively, were markedly upregulated (Fig. 4g). Furthermore, it is clearly seen in the COG classification statistics that the stimulation of C-T@Ti_3_C_2_ + US might cause a more significant effect on the metabolism of substances in bacteria, such as nucleotide transport and metabolism, carbohydrate transport and metabolism and lipid transport and metabolism, which is particularly evident in the protein-related pathway (amino acid transport and metabolism and translation, ribosomal structure and biogenesis).

### In vitro and in vivo biocompatibility of the C-T@Ti_3_C_2_ nanosheets

**Fig. 5:**
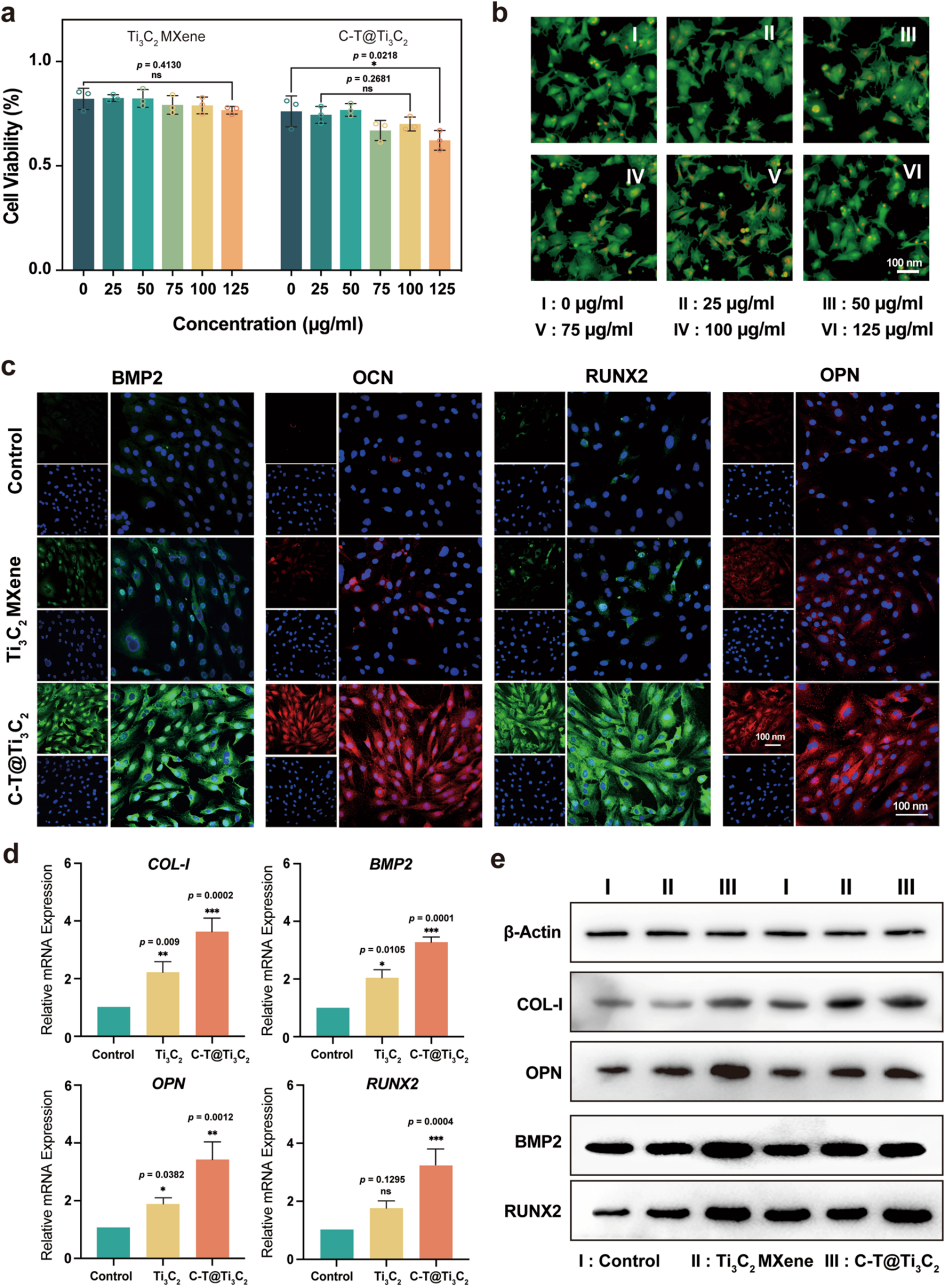
In vitro evaluation of C-T@Ti_3_C_2_ nanosheets for promoting osteogenesis. **a** Viability of rat bone marrow mesenchymal stem cells after incubation with different concentrations of C-T@Ti_3_C_2_ nanosheets (n = 3 for each group). **b** CLSM of AM/PI-stained rBMMSCs after various concentrations of C-T@Ti_3_C_2_ nanosheets. **c** CLSM images of the expression of BMP2, OCN, RUNX2 and OPN (at Day 14) in rBMMSCs after different treatments. A representative image of three replicates from each group is shown. **d** Relative mRNA levels of osteogenic genes (COL-I, BMP2, OPN and RUNX2). **e** Representative Western blots of total COL-I, OPN, BMP2, RUNX2 and β-actin after induction for 14 days in rBMMSCs. Data are presented as the mean ± SD, and statistical significance was calculated using the two-tailed t test, ^*^P < 0.05, ^**^P < 0.01, ^***^P < 0.001

To demonstrate the osteogenic ability of C-T@Ti_3_C_2_ nanosheets, rat bone marrow mesenchymal stem cells (rBMSCs) were chosen to evaluate the biocompatibility and osteogenic differentiation of C-T@Ti_3_C_2_ in vitro. The rBMSCs (1 × 10^6^/mL) were seeded with different concentrations of C-T@Ti_3_C_2_ in a 96-well plate and cultured for one day. The cell viabilities in this plate were then detected by using the Cell Counting Kit-8 (CCK-8) assay (Fig. 5a). Additionally, the excellent biocompatibility of C-T@Ti_3_C_2_ nanosheets was confirmed by a Calcein/PI Cell Viability/Cytotoxicity Assay Kit with Calcein-AM (green, live cells) and PI (red, dead cells). The fluorescent images of rBMSCs treated with C-T@Ti_3_C_2_ nanosheets showed intense green fluorescence, further validating the negligible cytotoxicity of C-T@Ti_3_C_2_ (Fig. 5b).

In addition, we performed in vivo systemic toxicity studies by administering 100 μL of C-T@Ti_3_C_2_ (100 μg mL^−1^) or an equivalent amount of saline into the tail vein of mice (n = 3). The routine blood parameters, including red blood cells (RBCs), mean corpuscular haemoglobin (MCH), haemoglobin (HGB), mean corpuscular haemoglobin concentration (MCHC), mean corpuscular volume (MCV), mean platelet volume (MPV), number of neutrophils (GRAN), red cell distribution width (RDW), number of lymphocytes (LYMPH), haematocrit (HCT) and platelets (PLT), were within normal limits and were not significantly different in either group. In addition, biochemistry parameters, including alanine transaminase (ALT), aspartate transaminase (AST), alkaline phosphatase (ALP), urea nitrogen (BUN), and creatinine (CREA), did not show any significant abnormalities (Additional file 1: Fig. S9). In addition, we performed H&E staining with the important organs of the mice, and no obvious difference was observed between the two groups; all the tissues were normal (Additional file 1: Fig. S10). All the data indicated that C-T@Ti_3_C_2_ showed good biocompatibility and low toxicity.

### In vitro osteogenic performances of the C-T@Ti_3_C_2_ nanosheets

As shown in Fig. 5c, we further assessed the osteogenic capacity of C-T@Ti_3_C_2_ nanosheets in promoting the osteogenic differentiation of rBMSCs in vitro by immunofluorescence staining. The expression levels of osteogenesis-related factors, morphogenetic protein-2 (BMP2), osteocalcin (OCN), runt-related transcription Factor 2 (RUNX2), and osteopontin (OPN) were detected by the fluorescence intensity. Obviously, the C-T@Ti_3_C_2_ group showed stronger fluorescence signals than that of the other groups. Encouraged by this, RT‒PCR was selected to evaluate osteogenic differentiation at the mRNA level by measuring osteogenic genes, including COL-I, BMP2, OPN, and RUNX2. Compared to those in the other groups, the mRNA expression of cells was much higher at Day 14 (Fig. 5d). Subsequently, according to the results of the Western blot assay, we obtained similar results (Fig. 5e). In conclusion, all these results confirmed that the C-T@Ti_3_C_2_ nanosheets exhibited high biocompatibility and enhanced osteogenic capacity in promoting the osteogenic differentiation of rBMSCs in vitro.

It can be inferred that the alkaline environment inside the rBMSCs is not conducive to the induction of CDT in C-T@Ti_3_C_2_ nanosheets. SDT can be induced under US stimulation, thereby producing a small amount of ROS, but no significant effect can be seen on the function of rBMSCs. Furthermore, the reaction of CaO_2_ NPs with H_2_O to produce O_2_ promoted osteogenic performance combined with the prolonged release of Ca^2+^, which played a long-term role in promoting osteogenesis.

### In vivo evaluation of MRSA-infected skin wounds

**Fig. 6:**
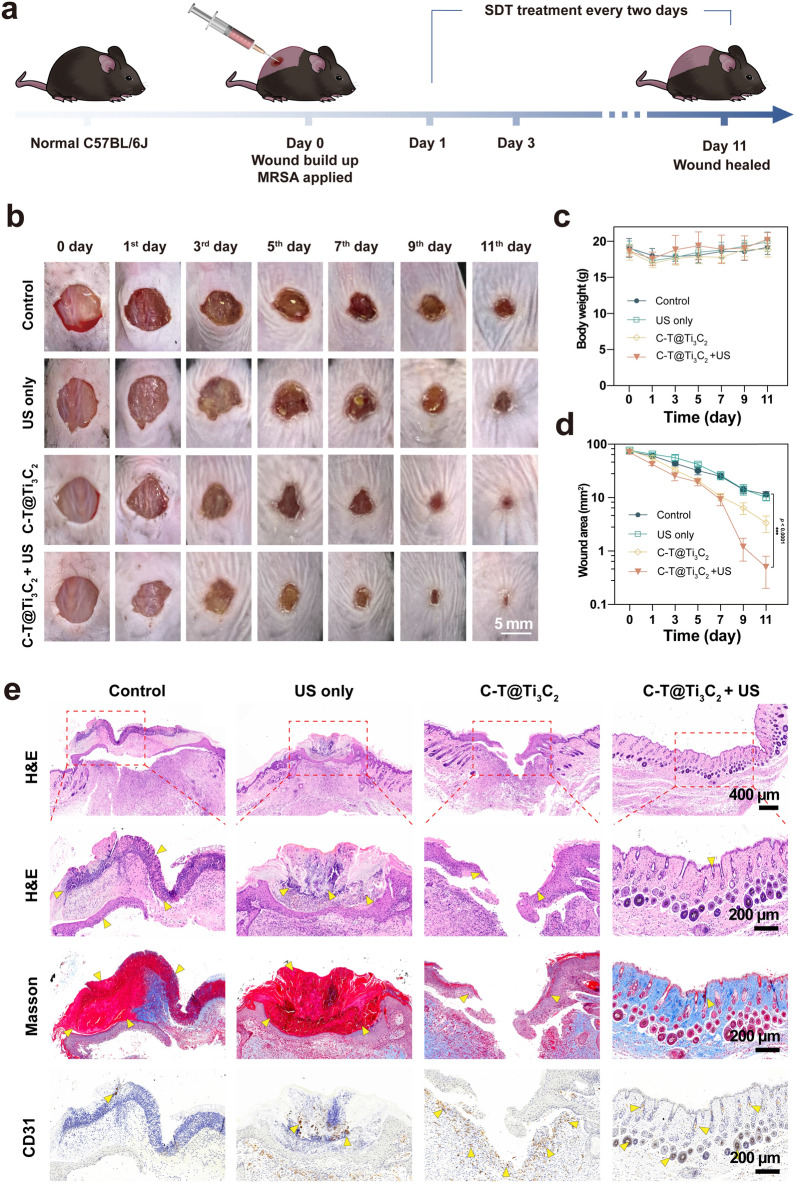
In vivo efficacy of the C-T@Ti_3_C_2_ nanosheets in the treatment of wound infection. **a** Schematic diagram of the creation of the wound infection model, treatment approach and the different timelines following treatment. **b** Representative digital photos of the *MRSA*-infected wounds treated with nonmaterial (control), US only, C-T@Ti_3_C_2_, and C-T@Ti_3_C_2_ + US on Days 0, 1, 3, 5, 7, 9 and 11. Statistics of **c** body weight and **d** wound area for each group of mice recorded after different treatments (n = 5). **e** Histological staining (H&E, Masson, and CD 31 immunohistochemical staining) of the representative wounds was performed from different groups after 11 days. Statistical significance was calculated using the two-tailed t test, ^*^P < 0.05, ^**^P < 0.01, ^***^P < 0.001

Based on the great biocompatibility and strong bactericidal effect, the therapeutic efficiency of the C-T@Ti_3_C_2_ nanosheets in vivo was evaluated in the skin defect model with an open and *MRSA*-infected wound in mice (Fig. 6a). The surfaces of the C-T@Ti_3_C_2_ nanosheets were modified by PVP molecules to improve biocompatibility and physiological stability in various physiological environments in this investigation [15, 20, 54]. An infectious wound was established in each mouse, and the mice were divided into four groups (n = 5), which were subsequently treated with nonmaterial (control group), US only, C-T@Ti_3_C_2_ and C-T@Ti_3_C_2_ + US. The wound healing processes in these groups were recorded for detailed analysis and statistics (Fig. 6b–d). Surgical procedures were standardized throughout the process. After different treatments, the wound areas in the C-T@Ti_3_C_2_ and C-T@Ti_3_C_2_ + US groups were smaller than those in the control and US only groups. This was facilitated by the antimicrobial activity of the C-T@Ti_3_C_2_ nanosheets, especially when combined with US stimulation (1 Wcm^−2^, 50% duty cycle, 1 MHz, 5 min), which resulted in the rapid release of large amounts of ROS; as a result, the ROS antagonized infection and promoted wound healing. Due to the burden of *MRSA*, the wound area of the control group with a final size of approximately 10 mm^2^ exhibited an ulcerated appearance and a reluctance to form scabs.

Wound samples from mice at Day 11 were subjected to different staining tests for further histological analysis (Fig. 6e), which included H&E staining, Masson staining and double immunofluorescence staining of vascular endothelial cell (CD31) markers. H&E staining showed that the C-T@Ti_3_C_2_ + US treatment resulted in the highest levels of epithelial and dermal regeneration, with a great reduction in the inflammatory response as well as follicle regeneration. The control and US only groups showed ruptured epithelial and dermal tissue, along with an infiltration of granulation tissue consisting of neutrophils and endothelial cells; these observations all indicated a severe inflammatory response to *MRSA* infection in the wound. Masson staining was used to detect collagen deposition and directional alignment, which are important in tissue regeneration. Masson staining showed massive collagen deposition and better skin recovery in the NIR group, while in the other groups, little or no collagen was observed. Double immunofluorescent labelling of CD31 was used to detect angiogenesis, which is a crucial indicator of the angiogenic process [55–57]. During the treatment, no significant difference in the body weight of the mice was observed, and important organs were stained by H&E staining to confirm that the C-T@Ti_3_C_2_ nanosheets did not cause side effects. These macroscopic and microscopic observations suggest that C-T@Ti_3_C_2_ nanosheets activated SDT, which in combination with CDT is highly effective in the treatment of wound infections caused by US-induced *MRSA*.

### In vivo evaluation of bone-tissue regeneration with MRSA injection

**Fig. 7:**
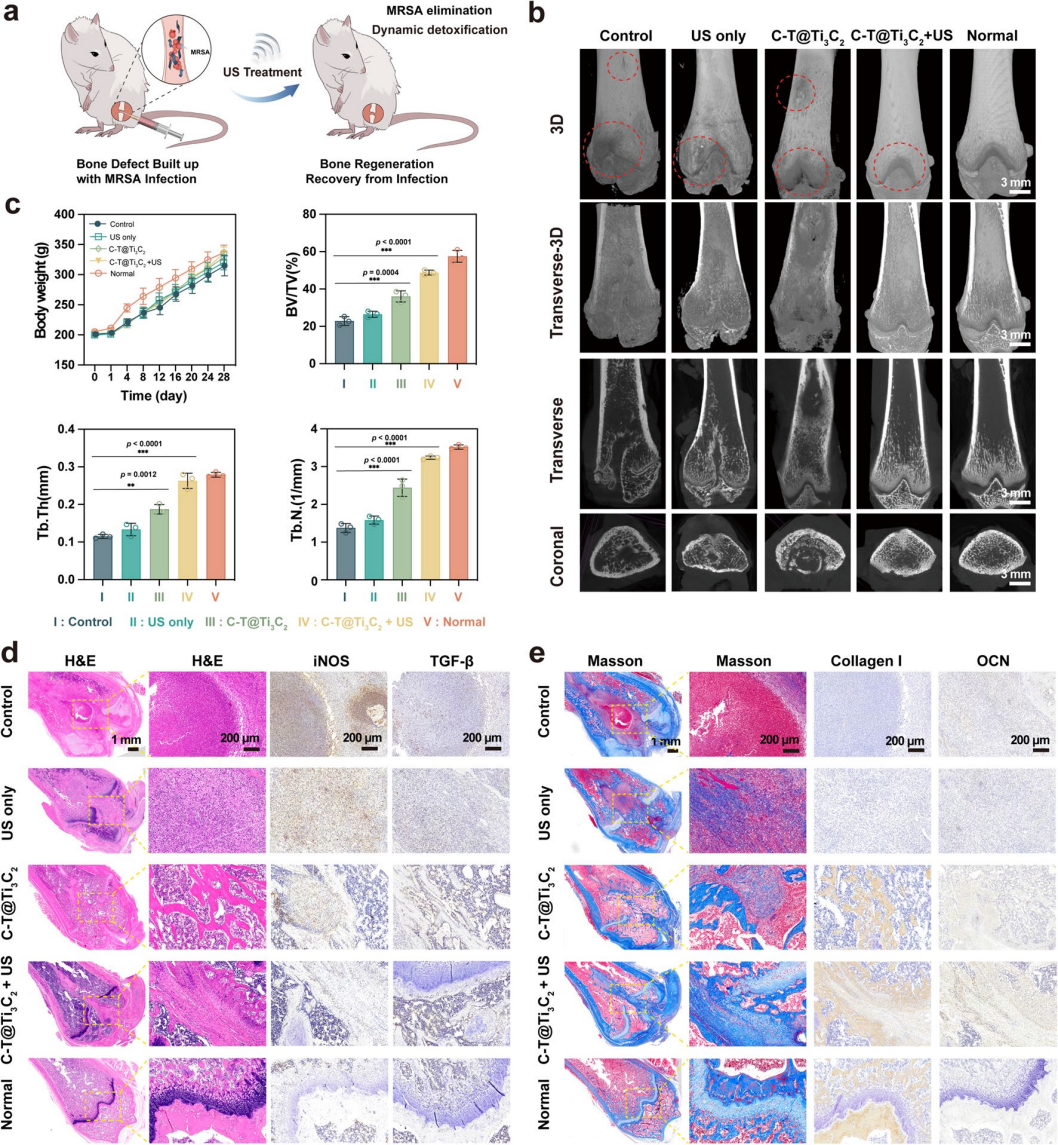
In vivo eradication of bone defects with a *MRSA* injection. **a** Schematic illustration of the *MRSA*-injected bone defect model. **b** Coronal, transverse, transverse (3D) and 3D micro-CT images in different groups after 4 weeks of treatment. **c** Statistics of body weight and quantitative analysis of BV/TV, Tb. Th and Tb. N after 4 weeks. **d**, **e** H&E, Masson, iNOS, TGF-**β**, Collagen I, and OCN staining images in different groups after surgery for 4 weeks. The statistical significance was calculated using a two-tailed t test, ^*^P < 0.05, ^**^P < 0.01, ^***^P < 0.001

The excellent biocompatibility and osteogenic performance of C-T@Ti_3_C_2_ nanosheets prompted us to establish a bone defect model with *MRSA* injection in male Sprague Dawley (SD) rats and to assess the antibacterial efficacy and bone regeneration ability of C-T@Ti_3_C_2_ in vivo (Fig. 7a). A circular bone defect was formed by drilling a hole in the lower femur of the rat with a bone drill and injecting 50 μL of *MRSA* suspension (10^7^ CFU mL^−1^). Thereafter, all the rats were randomly divided into five groups, and different treatments were administered to the indicated group. Then, the muscle and skin were sutured. Femurs were collected from rats at 14 and 28 days postoperatively and examined for bone defect repair and tissue regeneration via micro-CT scanning (Fig. 7b). Micro-CT results showed *MRSA*-induced cavitation in the femoral structures of the control, US only and C-T@Ti_3_C_2_ groups at 28 days postoperatively, but the C-T@Ti_3_C_2_ group exhibited enhanced bone repair compared with that of the other groups. After combining US, the femoral specimens exhibited more new bone tissue (Fig. 7b and Additional file 1: Fig. S12), and the defect area was markedly reduced. The bone volume to total volume (BT/TV), trabecular thickness (Tb. Th) and trabecular number (Tb.N.) were much higher for the group treated with C-T@Ti_3_C_2_ + US than for the other three groups, implying the superior antimicrobial effect and osteogenic properties of C-T@Ti_3_C_2_ (Fig. 7c and Additional file 1: Fig. S13). The changes in body weight of the rats were observed for over 4 weeks. A healthy trend of weight gain was observed among all groups, indicating that the inflammatory infection was confined to the local area and caused little alteration in the rats' general health (Fig. 7c).

H&E staining was performed to detect the degree of inflammation in bone tissues surrounding the defect sites (Fig. 7d). After 2 or 4 weeks of treatment, many inflammatory cells, including lymphocytes and neutrophils, were observed in the control and US groups, whereas few inflammatory cells infiltrated the C-T@Ti_3_C_2_ and C-T@Ti_3_C_2_ + US groups. Similarly, Masson staining images showed a marked reduction in the inflammatory response and elevated collagen deposition in bone tissues treated with C-T@Ti_3_C_2_ + US. To investigate the immune microenvironment of infected bone tissue, immunohistochemical staining of macrophages was performed. We found the lowest expression of inducible nitric oxide synthase (iNOS) and the highest expression of transforming growth factor-β (TGF-β) in the C-T@Ti_3_C_2_ + US group. This was because macrophages tended to polarize towards M2 due to the elimination of infection, which facilitated bone regeneration (Fig. 7d and Additional file 1: Fig. S14). In contrast, the control and US-only groups tended to polarize macrophages towards M1 due to persistent infection expression [58]. To further evaluate the osteogenic properties of C-T@Ti_3_C_2_ nanosheets in promoting bone regeneration, we also performed immunohistochemical staining of the femur to detect osteogenesis-related indicators. Masson staining showed that after C-T@Ti_3_C_2_ + US treatment, a larger amount of collagen was produced compared to that of the other groups. As shown in Fig. 7e and Additional file 1: Fig. S15, higher expression of Collagen I and OCN was detected in the C-T@Ti_3_C_2_ + US group, proving the excellent osteogenesis performance of C-T@Ti_3_C_2_ combined with US. HE staining of the major organs, including the heart, liver, spleen, lungs and kidneys, was performed after 2 and 4 weeks. No signs of organ damage was observed, as shown in the Supplementary Figs. 16 and 17, indicating that the prepared material was not histologically toxic. These results confirm that C-T@Ti_3_C_2_ nanosheets feature desirable antimicrobial and osteogenic properties under US stimulation, especially in deep tissue infections, and can counteract *MRSA* to alleviate the erosion of bone tissue and promote bone tissue regeneration, especially hyaline cartilage.

## Conclusions

In summary, we engineered C-T@Ti_3_C_2_ nanosheets with both sonodynamic and chemodynamic features for achieving effective antibacterial and bone-tissue regeneration. CaO_2_ NPs initially reacts in the weak acid environment to release large amounts of H_2_O_2_ immediately via the catalytic Fenton reaction to produce ·OH for inducing bacterial death. Spontaneously formed TiO_x_ by in situ oxidation of Ti_3_C_2_ MXene for use as an SDT sonosensitizer to produce ^1^O_2_ which cooperated with ·OH to enhance antibacterial. Sequently, Ca^2+^ generated by CaO_2_ were deposited to promote bone regeneration. Both in vitro and in vivo results validate that C-T@Ti_3_C_2_ nanosheets has not only the significantly ROS production but outstanding sono/chemo-dynamic effect, which leads to the satisfactory bacteria-growth inhibition outcome and the promotion of bone regeneration with high biocompatibility and biosafety. Our research presented a multifunctional nanocatalytic reaction system that might provide a potential instrument for the clinical treatment of infective bone defect or prosthetic joint infections in the clinic.

## Methods

### Materials and reagents

Layered titanium aluminium carbide (Ti_3_AlC_2_, 400 mesh powders with 98% metals basis) was purchased from Adamas-beta Inc. (Shanghai, China). Calcium hydroxide (Ca(OH)_2_), tetramethylammonium (TMAOH) hydroxide solution (25%), 3,3ʹ,5,5ʹ-tetramethylbenzidine dihydrochloride hydrate (TMB), and (3-aminopropyl)triethoxysilane (APTES) were obtained from Macklin Inc. (Shanghai, China). 1,3-Diphenylisobenzofuran (DPBF) was kindly provided by Adamas-beta Inc. (Shanghai, China). Polyvinyl pyrrolidone (PVP), penicillin‒streptomycin-amphotericin B solution, and 2,7-dichlorofluorescein diacetate (DCFH-DA) were purchased from Solarbio Co., Ltd. (Beijing, China). The Live & Dead Bacterial Staining Kit was purchased from YEASEN Biotech Co., Ltd. (Shanghai, China). Cell Counting Kit-8 (CCK-8) was obtained from Biosharp (Anhui, China). Calcein/PI Cell Viability/Cytotoxicity Assay Kit, radioimmunoprecipitation assay (RIPA) buffer and 2-(4-amidinophenyl)-6-indolecarbamidine dihydrochloride (DAPI) were from Beyotime Institute of Biotechnology (Shanghai, China). 2,2,6,6-Tetramethylpiperidine (TEMP) was obtained from Aladdin Ltd. (Shanghai, China). Hydrogen peroxide (H_2_O_2_) was purchased from Sigma‒Aldrich Trading Co., Ltd. (Shanghai, China). Ammonia water (NH_3_-H2O, 30%), hydrogen fluoride aqueous solution (40%), dimethyl sulfoxide (DMSO) and alcohol were provided by Sinopharm Chemical Reagent Co., Ltd. (Shanghai, China). Anti-BMP2 antibody (#ab284387), anti-collagen I (#ab284387), anti-osteocalcin antibody (#ab133612), anti-RUNX2 antibody (#ab236639) and anti-beta actin antibody (#ab8226) were obtained from Abcam Inc. (Cambridge, MA, USA). Osteopontin antibody (#AF0227) was purchased from Affinity Biosciences (Jiangsu, China). Foetal bovine serum (FBS), phosphate buffered saline (PBS) and Dulbecco’s modified Eagle’s medium nutrient mixture F-12 (DMEM/F-12) were provided by Gibco. Paraformaldehyde was purchased from Wuhan Servicebio Technology Co., Ltd. (Wuhan, China). Deionized (DI) water with a resistivity of 18.2 Ω was used throughout this project.

### Synthesis of Ti_3_C_2_ (MXene) and CaO_2_-TiO_x_@Ti_3_C_2_ (C-T@Ti_3_C_2_) nanosheets

To synthesize single- and few-layered Ti_3_C_2_ MXenes, 60 mL of HF (40 wt%) solution was carefully mixed with 4G Ti_3_AlC_2_ MAX in an autoclave with a Teflon liner for 72 h at room temperature (RT) to remove the Al layer. Then, the precipitate was collected by high-speed centrifugation (20,000 rpm for 15 min) and washed several times with ethanol and DI water until the pH of the supernatant reached 6.0. The bulky precipitate was obtained and stirred with 60 mL of a TMAOH aqueous solution for three days at RT. After centrifugation (4000 rpm, 15 min), single- and few-layered Ti_3_C_2_ MXenes were collected from the colloidal supernatant. PVP (6 g) was dissolved in 12 mL of DI water. Then, Ca(OH)_2_ (0.6 g) was added and stirred for 20 min. Then, 4 mL H_2_O_2_ (4.0 mL) was added dropwise with vigorous stirring afterwards. After 15 min, stable CaO_2_ NPs formed on the Ti_3_C_2_ MXene. Finally, the product was obtained through centrifugation and washed with DI water and ethanol. Since the surface potential of Ti_3_C_2_ MXene was negative, surface modification was performed. In short, APTES (100 μL) was added to Ti_3_C_2_ MXene (1 mg mL^−1^, 20 mL) and stirred at 50 °C for 24 h to form Ti_3_C_2_-NH_2_. To obtain CaO_2_-TiO_x_@Ti_3_C_2_ nanosheets, Ti_3_C_2_-NH_2_ composite nanosheets were stirred with CaO_2_ NPs in ethanol for 24 h and then centrifuged to obtain CaO_2_-TiO_x_@Ti_3_C_2_ (C-T@Ti_3_C_2_) composite nanosheets. The resulting C-T@Ti_3_C_2_ nanosheets were stored at 4 °C for further experiments.

### Material characterization

Transmission electron microscopy (TEM) images were obtained on a JEM-2100F transmission electron microscope at an acceleration voltage of 200 kV. Scanning electron microscopy (SEM) images and bio-SEM images were acquired on an SU8220 microscope (HITACHI, Japan) and Regulus 8100 (HITACHI, Japan), respectively. High-resolution STEM, HAADF/ABF-STEM images, elemental mapping and relative EDS analysis were carried out on a JEM-ARM 300F Grand ARM (JEOL Company Ltd., Japan) at 80 kV with two spherical aberration correctors. XPS measurements were performed on ESCAlab250 electron spectrometers (Thermal Fisher VG, USA). X-ray diffraction analysis (XRD) was performed by a Rigaku automated multipurpose X-ray diffractometer (Smartlab, Rigaku Co. Ltd., Tokyo, Japan). Atomic force microscopy (AFM) was performed by a Bruker Dimension Icon (Bruker, German). Raman spectroscopy was performed using an inVia confocal Raman microscope (Reinshaw, UK). Raman spectra were recorded by a Horiba Jobin Y’ von (LabRAM HR) micro-Raman spectrometer (Horiba, USA). Dynamic light scattering (DLS) and zeta potential examinations were obtained from the Malvern Nano-ZS90 Zetesizer (Malvern Instrument Ltd., US). UV‒vis–NIR absorption spectra were obtained on a UV-1800 spectrometer (MAPADA, China). The ^1^O_2_ and ·OH production were detected using a JEOL-FA200 ESR spectrometer (JEOL Company Ltd., Japan). Confocal laser scanning microscopy (CLSM) images were captured by a high-speed confocal platform (Andor, UK). Tissue sections were observed by Olympus VS120 (Olympus Company, Japan).

### Surface modification of the CaO_2_-TiO_x_@Ti_3_C_2_ nanosheets

The surface of CaO_2_-TiO_x_@Ti_3_C_2_ was modified to help stabilize the nanosheet in a physiological environment. Ten milligrams of CaO_2_-TiO_x_@Ti_3_C_2_ nanosheets were dispersed in an aqueous PVP solution (1 mg mL^−1^, 20 mL) at 50 °C and stirred for 8 h. After centrifugation and washing with water and ethanol, the CaO_2_-TiO_x_@Ti_3_C_2_ composite nanosheets were obtained and stored at 4 °C for the following in vivo investigation.

### Detection of ROS generation

To detect ROS generated by sonodynamic therapy (SDT), 1 mL of C-T@Ti_3_C_2_ was mixed with 20 μL of DPBF (1 mg mL^−1^ in ethanol) along with DI water to form a 3 mL reaction system. The absorbance changes in DPBF were observed at the characteristic peak (416 nm) after ultrasonic stimulation (1 Wcm^−2^, 50% duty cycle, 1 MHz) for different times ranging from 0 to 8 min. Similarly, TMB (20 mg mL^−^1 in DMSO) was utilized to detect the generation of hydroxyl radicals (·OH) after different reaction times (from 0 to 30 min) by monitoring the increase in absorbance at 662 nm with a UV‒vis spectrometer. Furthermore, ROS generation by ultrasonically activated C-T@Ti_3_C_2_ was recorded by DCFH-DA, fixed to 3 ml with DI water and subjected to different treatments. The fluorescence intensity of DCF was detected by a FLS1000 spectrofluorometer (Edinburgh Instrument, US) with an excitation wavelength of 488 nm.

### Quantitative generation of ^1^O_2_ and OH

The synthesized C-T@Ti_3_C_2_ nanosheets and Ti_3_C_2_ MXene were detected for ^1^O_2_ and •OH production by ESR spectroscopy at room temperature. In brief, TEMP and DMPO, as spin trapping agents for singlet oxygen and hydroxyl radicals, respectively, were incubated with DI water, Ti_3_C_2_ MXene and C-T@Ti_3_C_2_ nanosheets at room temperature and ultrasonicated (1 Wcm^−2^, 50% duty cycle, 1 MHz) for 5 min. Afterwards, the ESR spectra were recorded for three samples.

### Antibacterial experiment in vitro

The antimicrobial efficiency of C-T@Ti_3_C_2_ nanosheets and Ti_3_C_2_ MXene was evaluated through a spread plate assay. Escherichia coli (*E. coli*, ATCC 35401) was used as the gram-negative bacterial strain, while Staphylococcus aureus (*S. aureus*, ATCC 6538) and methicillin-resistant Staphylococcus aureus (*MRSA*, ATCC 43300) represented the gram-positive bacterial model. All of them were purchased from BeNa Culture Collection Co., Ltd. (Beijing, China). They were cultured in sterile Luria–Bertani (LB) medium at 37 °C in an aerophilic environment for 12 to 16 h. The diluted bacterial suspension (10^9^ CFU mL^−1^) was utilized for the following experiments after testing the absorbance at 600 nm by a microplate reader. The bacterial suspension was diluted with sterile DI water to 10^6^ CFU mL^−1^ and incubated with C-T@Ti_3_C_2_ nanosheets at different concentrations (0, 25, 50, 150 and 200 μg mL^−1^) at 37 °C for 4 h. Batch assays were performed with continuous orbital shaking at 150 rpm. The resulting bacterial suspension (20 µL) was further diluted and spread on prepared LB agar plates by bacterial spread rods with three replicates per sample. The plates were incubated for an additional 12–16 h at 37 °C, and bacterial viability was measured by counting the numbers of bacterial colonies. To further validate the sonodynamic effect of the nanosheets, four experimental groups were set up, and two of the groups were added to coculture the bacterial suspension with the C-T@Ti_3_C_2_ nanosheets (the remaining two groups were not treated) for 1 h. The bacterial mixture from one of the blank groups and one of the C-T@Ti_3_C_2_ groups was also sonicated (1 Wcm^−2^, 50% duty cycle, 1 MHz) for 5 min (US only group and C-T@Ti_3_C_2_ + US group). The resulting bacterial suspension was diluted and spread evenly on agar plates and then incubated for 16 h at 37 °C.

For live/dead staining, samples with *S. aureus*, *MRSA* and *E. coli* were incubated at 37 °C for 12–16 h. They were then immersed in a Live/Dead Baclight Viability Kit of SYTO9 (green, live bacteria) and propidium iodide (PI, red, dead bacteria) for 15 min in the dark and then rinsed with PBS. Finally, photographs were obtained with a confocal fluorescence microscope (High speed confocal platform Dragonfly 200, US).

To further illustrate the antibacterial activity, the bacteria were examined morphologically using SEM. The samples were fixed in 2.5% glutaraldehyde for 6 h. Subsequently, the cells were washed three times with PBS, followed by dehydration with an ethanol gradient (25, 50, 75, 90 and 100 v/v%) for 15 min. Finally, the samples were observed under SEM.

### Detection of intercellular ROS

Fluorescence imaging was performed to investigate the level of intercellular ROS. A 200 μL *MRSA* suspension (10^9^ CFU mL^−1^ in LB medium) was placed in 96-well plates with different treatments (control, US only, Ti_3_C_2_ MXene, Ti_3_C_2_ MXene + US, C-T@Ti_3_C_2_ and C-T@Ti_3_C_2_ + US). Next, the samples were coincubated with 10 μL of DCFH-DA (10 μM) in DMSO solution for 30 min at room temperature and washed with sterilized DI water to remove excess dye. The samples were then observed under a confocal fluorescence microscope as mentioned above.

### RNA sequence analysis for MRSA

Methicillin-resistant Staphylococcus aureus (*MRSA*, ATCC 43300) at a concentration of 10^9^ CFU mL^−1^ was treated with C-T@ Ti_3_C_2_ nanosheets for 5 min under ultrasonication (1 Wcm^−2^, 50% duty cycle, 1 MHz). RNAseq analysis was performed by technical staff at Hangzhou Kaitai Biolab. Sequencing was carried out on a MiSeq instrument (Novaseq 6000® Sytem, Illumina Inc.). Quality examination was analysed by an Agilent Bioanalyzer (Qseq100 DNA Analyser, Bioptic Inc.) and real-time PCR (LightCycler® 96, Roche Inc.). Fastp was used to process raw data (https://github.com/OpenGene/fastp). Gene expression was calculated by Stringtie (https://ccb.jhu.edu/software/stringtie), and genes with FDR < 0.05 and |log2FC|≥ 1 were considered differentially expressed genes (DEGs). Additionally, KEGG pathway enrichment analysis was performed using the R language to detect paths associated with DEGs.

### In vitro cytotoxicity evaluation

The surfaces of Ti_3_C_2_ MXene and C-T@Ti_3_C_2_ were modified by PVP molecules to improve biocompatibility and physiological stability for all cellular and animal experiments in this investigation. Primary rat bone marrow mesenchymal stem cells (rBMMSCs) were obtained from SD rats and further cultured at 37 °C in Dulbecco's Modified Eagle Medium (DMEM), 5% foetal bovine serum (FBS) and 1% penicillin streptomycin, 95% humidity and 5% CO_2_. The rBMMSCs were incubated with C-T@Ti_3_C_2_ nanoworks at gradient concentrations (0, 25, 50, 75, 100 and 125 μg mL^−1^) for 24 h. Cell viability was tested by a microplate reader (Infinite m nano 2022657S, Tecan Spark) using the Cell Counting Kit-8 (CCK8) assay. For the live/dead staining assay, rBMMSCs were cultured in confocal dishes and incubated with C-T@Ti_3_C_2_ nanoworks at different concentrations (0, 25, 50, 75, 100 and 125 μg mL^−1^) separately after washing with PBS twice. Next, the dishes were placed in the cell incubator for another 8 h. Then, the cells were stained with Calcein-AM/PI for 20 min before washing with PBS twice. The ultimate images were observed through CLSM.

### Fluorescence imaging of rBMMSCs

The rBMMSCs were fixed on 24-well plates with 4% paraformaldehyde for 15 min and then washed 3 times with PBS and 0.2% Triton X-100 (W/V) in PBS for 15 min. The cells were then blocked with 5% bovine serum albumin (BSA) for 30 min. The cells were incubated for 12 h at 4 °C with primary antibodies (BMP2, OCN, OPN and RUNX2), followed by a one-hour incubation with secondary antibodies. In addition, the nuclei of the cells were stained with DAPI. Images were captured with CLSM.

### Real-time quantitative polymerase chain reaction (RT‒qPCR) analysis

The mRNA transcript levels of osteogenic-specific genes were detected via real-time quantitative reverse transcription PCR (RT‒qPCR). The osteogenic differentiation of rBMMSCs treated with Ti_3_C_2_ MXene or C-T@Ti_3_C_2_ was assessed by detecting the mRNA expression of osteogenic genes, including collagen type I (COL-I), morphogenetic protein-2 (BMP2), osteomucin (OPN) and runt-related transcription Factor 2 (RUNX2). After osteogenic induction for 14 days, total RNA was extracted from stimulated rBMMSCs using TRIzol reagent, and the purified RNA was reverse transcribed into complementary DNA (cDNA) using PrimeScript RT Master Mix.

### Western blot analysis

After 14 days of different treatments (control, Ti_3_C_2_ MXene and C-T@Ti_3_C_2_), the cells were collected on ice and washed with ice-cold PBS solution twice. To prepare the cell lysates, the samples were resuspended in RIPA buffer containing protease inhibitors. After incubation on ice for 30 min, the cell lysates were clarified by centrifugation (10,000 rpm, 10 min) at 4 ℃ to remove debris, and the protein content was detected via the BCA protein assay kit. Equal amounts of extracts were diluted in sample buffer, subjected to 10% sodium dodecyl sulfate‒polyacrylamide gel electrophoresis (SDS‒PAGE) and transferred to a polyvinylidene difluoride (PVDF) membrane. Next, nonspecific binding was blocked with 5% (w/v) nonfat dry milk in Tris-buffered saline containing 0.1% Tween-20 (TBST) for 1 h at room temperature. Immunoblotting was carried out with antibodies. The samples were immunoblotted with the following primary antibodies: β-actin (1:1000 dilution), BMP2 (1:1000 dilution), RUNX2 (1:1000 dilution), COL-I (1:1000 dilution) and OPN (1:1000 dilution) at 4 °C overnight. After washing three times with TBST, the PVDF membrane was incubated with the secondary antibody for 1 h at room temperature. The target proteins were detected using a Luminescent Immunoanalyzer (MiniChemi610, Beijing). Anti-β-actin was used as a reference to monitor equal protein loading in the amounts of protein among samples.

### In vivo biocompatibility evaluation

Six C57BL/6JNifdc mice (8 weeks old, male, 20 g in weight) were purchased from Vital River Laboratory Animal Technology in Beijing. The ethics of animal experiments were approved by the Ethics Committee on animal experiments of Shandong University Qilu Hospital (Approval number: DWLL-2021-076). Six mice were randomly divided into the following groups: the control group was administered 100 μL of physiological saline through tail vein injection, and the other group was treated with 100 μL of C-T@Ti_3_C_2_ nanosheets (at a dose of 20 mg/kg) for 14 days of observation. After 14 days, the mice were euthanized, and major tissues (heart, liver, spleen, lung and kidney) along with blood samples were obtained for subsequent H&E staining for pathological evaluation. Routine blood and biochemical tests (RBC, MCH, HGB, MCHC, MCV, MPV, GRAN, RDW, LYMPH, HCT, PLT, ALT, AST, ALP, BUN, and CREA) were carried out using automatic blood cell analysers (BC-2800vet, Maydeal and Chemray 240, Rayto).

### In vivo wound healing model in mice

All animal surgical procedures were approved by the Ethics Committee on Animal Experiments of Shandong University Qilu Hospital in China (Approval No. DWLL-2021-076). The mouse wound defects model was operated on male C57BL/6JNifdc mice (8 weeks old, male, 20 g in weight), which were provided by Beijing Vital River Laboratory Animal Technology Co., Ltd. The *MRSA* (ATCC 43300) used for the infection model was in the mid-exponential growth phase at 10^7^ CFU mL^−1^. Mice were anaesthetized using isoflurane by airway inhalation at a rate of 600 mL/min for 5 min, then their backs were depilated and disinfected with iodophor and 75 v/v% alcohol. A circular wound 8–10 mm in diameter was made on the back, and twenty mice were randomly divided into four groups (control, US only, C-T@Ti_3_C_2_ and C-T@Ti_3_C_2_ + US). In the control and US only groups, we added approximately 60 µL to the wounds, while in the other two groups, we added equal amounts of C-T@Ti_3_C_2_ nanosheets. In particular, the wounds of the US only group and C-T@Ti_3_C_2_ + US group were implemented with ultrasound treatment (1 Wcm^−2^, 50% duty cycle, 1 MHz) for 5 min each time every two days. The body weight and wound size were recorded at 1, 3, 5, 7, 9, and 11 days. All mice were sacrificed after 12 days, and the granulation tissue from the wounds and the major organs (heart, liver, spleen, lung, and kidney) were excised for further histological analysis. Mouse tissue sections were observed using an Olympus VS120 panoramic digital section scanning microscope (Olympus Company, Japan).

#### In vivo bone defect model with MRSA infection

Sprague Dawley rats (6 weeks old, male, 200 g in weight) were provided by Beijing Vital River Laboratory Animal Technology Co., Ltd. The surgeries on animals were performed under the approval of the Ethics Committee on Animal Experiments of Shandong University Qilu Hospital (Approval No. DWLL-2021-077). Fifteen rats were anaesthetized, shaved around the knee joint and disinfected with iodophor and alcohol, and they were randomly divided into five groups (normal, control, US only, C-T@Ti_3_C_2_ and C-T@Ti_3_C_2_ + US). The skin was incised with sterilized surgical instruments, the muscle layer was opened, and the femur was exposed. Then, a 1.5–2 mm diameter hole was formed in the lower end of the femur with a bone drill. The suspension of *MRSA* (ATCC 43300) was injected into the bone marrow of four groups (control, US only, C-T@Ti_3_C_2_ and C-T@Ti_3_C_2_ + US), and C-T@Ti_3_C_2_ nanoworks were added to the two groups (C-T@Ti_3_C_2_ and C-T@Ti_3_C_2_ + US), while an equal amount of saline was added to the other two groups (control and US only), and the wound was finally closed layer by layer. SDT treatments were performed every 2 days, with each rat treated at 1.5 Wcm^−2^, 50% duty cycle, 1 MHz, for 15 min. To assess the effectiveness of the treatment, five groups of rats were sacrificed at the second and fourth postoperative weeks, and the femur was removed to observe the condition of the bone tissue under micro-CT (SkyScan 1276, Bruker, Belgium). All rats were sacrificed after four weeks, and the bone tissue was excised for further histological analysis with H&E, iNOS, TGF-β dye, Masson, COL-I and OCN. In addition, the major organs (heart, liver, spleen, lung, and kidney) were collected for H&E staining evaluation. Images of tissue slices were captured by an Olympus VS120 panoramic digital section scanning microscope (Olympus Company, Japan).

### Statistical analysis

All the data in the experiments are presented as the mean ± standard deviation (SD) and were repeated at least three times. The significance of the data in this work was analysed by two-tailed t test and two-way analysis of variance test using GraphPad Prism 9.3 (Graph Pad Software Inc.). The tests were regarded as statistically significant with signs of *P < 0.05, **P < 0.01, and ***P < 0.001.

## Supplementary Information

**Additional file 1: Fig. S1.** Digital photographs of Ti_3_AlC_2_, Ti_3_C_2_ MXene, CaO_2_-PVP and CaO_2_-TiO_x_@Ti_3_C_2_ (C-T@Ti_3_C_2_). **Fig. S2.** SEM image of multilayered Ti_3_C_2_ MXene. **Fig. S3.** TEM, dark-field STEM images and corresponding element mappings (for Ti, C, Ca and O) of C-T@Ti_3_C_2_ nanosheets. **Fig. S4.** DLS analysis of CaO_2_-PVP in water. **Fig. S5.** DLS analysis for Ti_3_C_2_ MXene in water and saline. **Fig. S6.** UV‒vis absorption spectra of time-dependent DPBF degradation under US treatment. **Fig. S7.** Digital photos of spread plates containing *E. coli, S. aureus,* and *MRSA* with various treatments. **Fig. S8.** Fluorescence microscopy images of *MRSA* stained with DCFH-DA after various treatments for ROS detection (I: Control group, II: US only group, III: Ti_3_C_2_ MXene group, IV: Ti_3_C_2_ MXene + US group, V: C-T@Ti_3_C_2_ group, VI: C-T@Ti_3_C_2_ + US group). **Fig. S9.** Haematological index of C57BL/6JNifdc mice intravenously administered C-T@Ti_3_C_2_ nanosheets dispersion for 14 d (n = 3). Data are presented as the mean ± SD. Routine blood parameters included red blood cells (RBCs), mean corpuscular haemoglobin (MCH), haemoglobin (HGB), mean corpuscular haemoglobin concentration (MCHC), mean corpuscular volume (MCV), mean platelet volume (MPV), number of neutrophils (GRAN), red cell distribution width (RDW), number of lymphocytes (LYMPH), haematocrit (HCT) and platelets (PLT). Biochemistry parameters included alanine transaminase (ALT), aspartate transaminase (AST), alkaline phosphatase (ALP), urea nitrogen (BUN), and creatinine (CREA). **Fig. S10.** Histological assessments of the major organs (heart, liver, spleen, lung and kidney) of C57BL/6JNifdc mice after intravenous injections with C-T@Ti_3_C_2_ nanonetworks for 14 d. **Fig. S11.** Histological assessments of the major organs (heart, liver, spleen, lung and kidney) of C57BL/6JNifdc mice after different treatments in the MRSA-infected wound model. **Fig. S12.** Micro-CT images of femurs in different groups after 2 weeks of treatment. **Fig. S13.** Corresponding quantitative analysis showing BV/TV, Tb. Th and Tb.N. (n = 3) in different groups after surgery for 2 weeks. Data are presented as the mean ± SD. Statistical significance was calculated with a two-tailed t test, *P ≤ 0.05, **P ≤ 0.01, ***P ≤ 0.001. **Fig. S14.** Histological assessments (H&E staining, iNOS and TGF-β immunohistochemical staining) of bone tissue in different groups after 2 weeks of treatment. **Fig. S15.** Histological assessments (Masson staining, collagen I and OCN immunohistochemical staining) of the bone tissue in different groups after 2 weeks of treatment. **Fig. S16.** Histological assessments of the major organs (heart, liver, spleen, lung and kidney) of SD rats from the bone defect model with *MRSA* infection at 2 weeks. **Fig. S17.** Histological assessments of the major organs (heart, liver, spleen, lung and kidney) of SD rats from the bone defect model with *MRSA* infection at 4 weeks.

## Data Availability

All data generated or analysed during this study are included in this article.
