# Supplementary material for: Spontaneous formation of MXene-oxidized sono/chemo-dynamic sonosensitizer/nanocatalyst for antibacteria and bone-tissue regeneration
Source: J Nanobiotechnology. 2023 Jun 14;21:193. doi: 10.1186/s12951-023-01933-z (PMC10268399; doi:10.1186/s12951-023-01933-z)
Supplement: Supplementary file 1 — Additional file 1: Fig. S1. Digital photographs of Ti3AlC2, Ti3C2 MXene, CaO2-PVP and CaO2-TiOx@Ti3C2 (C-T@Ti3C2). Fig. S2. SEM image of multilayered Ti3C2 MXene. Fig. S3. TEM, dark-field STEM images and corresponding element mappings (for Ti, C, Ca and O) of C-T@Ti3C2 nanosheets. Fig. S4. DLS analysis of CaO2-PVP in water. Fig. S5. DLS analysis for Ti3C2 MXene in water and saline. Fig. S6. UV‒vis absorption spectra of time-dependent DPBF degradation under US treatment. Fig. S7. Digital photos of spread plates containing E. coli, S. aureus, and MRSA with various treatments. Fig. S8. Fluorescence microscopy images of MRSA stained with DCFH-DA after various treatments for ROS detection (I: Control group, II: US only group, III: Ti3C2 MXene group, IV: Ti3C2 MXene + US group, V: C-T@Ti3C2 group, VI: C-T@Ti3C2 + US group). Fig. S9. Haematological index of C57BL/6JNifdc mice intravenously administered C-T@Ti3C2 nanosheets dispersion for 14 d (n = 3). Data are presented as the mean ± SD. Routine blood parameters included red blood cells (RBCs), mean corpuscular haemoglobin (MCH), haemoglobin (HGB), mean corpuscular haemoglobin concentration (MCHC), mean corpuscular volume (MCV), mean platelet volume (MPV), number of neutrophils (GRAN), red cell distribution width (RDW), number of lymphocytes (LYMPH), haematocrit (HCT) and platelets (PLT). Biochemistry parameters included alanine transaminase (ALT), aspartate transaminase (AST), alkaline phosphatase (ALP), urea nitrogen (BUN), and creatinine (CREA). Fig. S10. Histological assessments of the major organs (heart, liver, spleen, lung and kidney) of C57BL/6JNifdc mice after intravenous injections with C-T@Ti3C2 nanonetworks for 14 d. Fig. S11. Histological assessments of the major organs (heart, liver, spleen, lung and kidney) of C57BL/6JNifdc mice after different treatments in the MRSA-infected wound model. Fig. S12. Micro-CT images of femurs in different groups after 2 weeks of treatment. Fig. S13. Corresponding quantitative [file 12951_2023_1933_MOESM1_ESM.docx]

**Additional Information**

**Spontaneous Formation of MXene-Oxidized Sono/Chemo-Dynamic Sonosensitizer/Nanocatalyst for Antibacteria and Bone-Tissue Regeneration**

Yang Yu^1,2,3^**^†^**, Houyi Sun^1^**^†^**, Qunshan Lu^1^, Junyuan Sun^1,3^, Pengfei Zhang^1,3^, Linran Zeng^4^, Krasimir Vasilev^5^, Yunpeng Zhao^1*^, Yu Chen^2*^, Peilai Liu^1*^

^1^Department of Orthopaedics, Qilu Hospital of Shandong University, Jinan 250012, P. R. China

^2^Materdicine Lab, School of Life Sciences, Shanghai University, Shanghai 200444, P. R. China

^3^Laboratory of Basic Medical Sciences, Qilu Hospital of Shandong University, Jinan 250012, P. R. China.

^4^The 1st Affiliated Hospital of Kunming Medical University, Kunming Yunnan 650032, P. R. China

^5^ College of Medicine and Public Health, Flinders University, Sturt Road, Bedford Park South Australia, 5042, Australia

**^†^** These authors contribute equally.

***Corresponding authors**

Yunpeng Zhao, Yu Chen, Peilai Liu, Emails: lwwzyp@email.sdu.edu.cn (Prof. Y. Zhao); 199362000205@email.sdu.edu.cn (Prof. P. Liu); chenyuedu@shu.edu.cn (Prof. Y. Chen)

**Authors’ emails**

Yang Yu, gkyy1998@mail.sdu.edu.cn

Houyi Sun, 15051514605@163.com

Qunshan Lu, [luqunshan1112@163.com](mailto:luqunshan1112@163.com)

Junyuan Sun, 17621778576@163.com

Pengfei Zhang, pengfeizhang@mail.sdu.edu.cn

Linran Zeng, 147794316@qq.com

Krasimir Vasilev, krasimir.vasilev@flinders.edu.au

Yunpeng Zhao, lwwzyp@email.sdu.edu.cn

Yu Chen, chenyuedu@shu.edu.cn

Peilai Liu, 199362000205@email.sdu.edu.cn

**Additional figures**

**
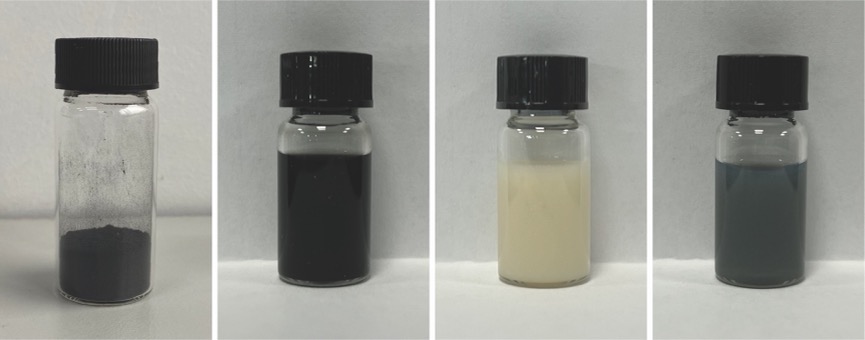
**

**Figure S1.** Digital photographs of Ti_3_AlC_2_, Ti_3_C_2_ MXene, CaO_2_-PVP and CaO_2_-TiO_x_@Ti_3_C_2_ (C-T@Ti_3_C_2_).


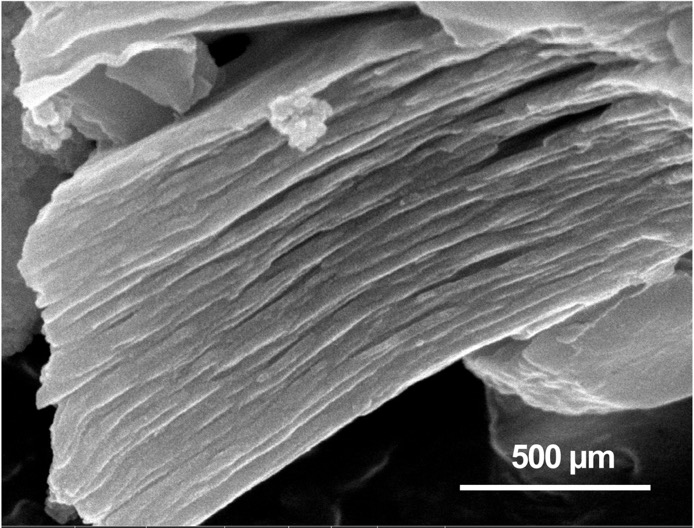


**Figure S2.** SEM image of multilayered Ti_3_C_2_ MXene.


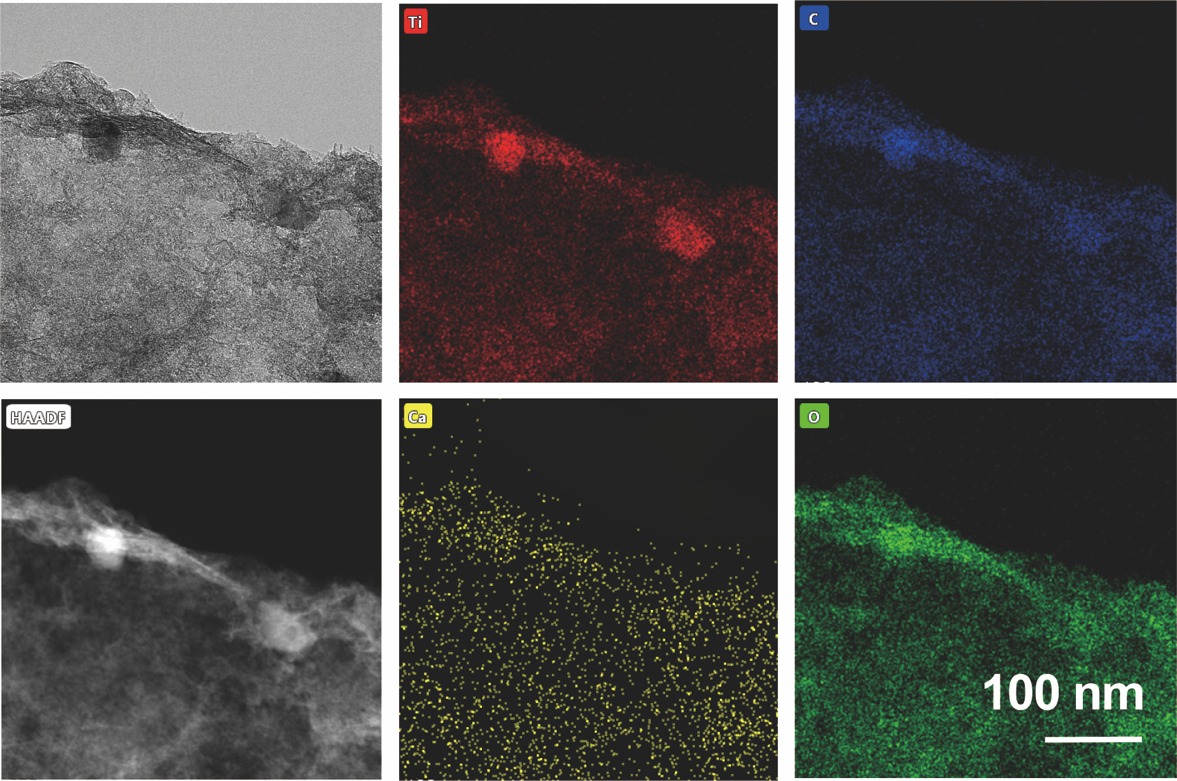


**Figure S3.** TEM, dark-field STEM images and corresponding element mappings (for Ti, C, Ca and O) of C-T@Ti_3_C_2_ nanosheets.


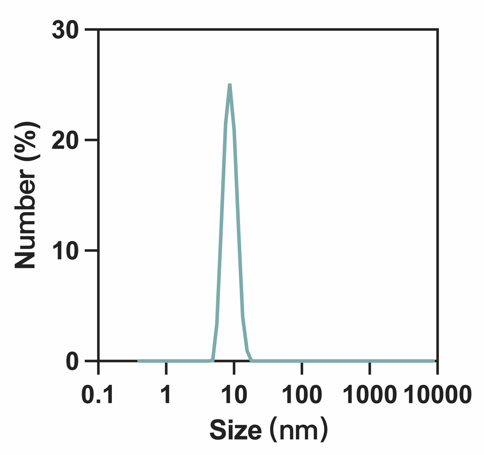


**Figure S4.** DLS analysis of CaO_2_-PVP in water.


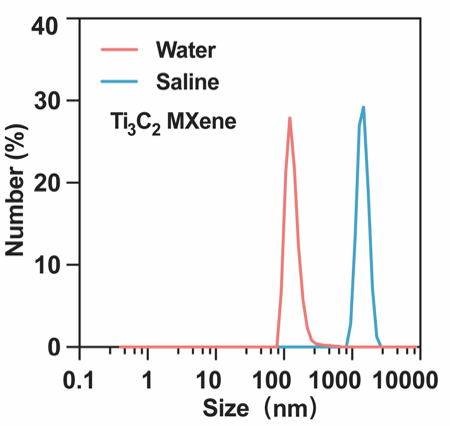


**Figure S5.** DLS analysis for Ti_3_C_2_ MXene in water and saline.


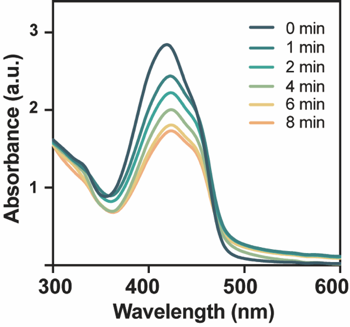


**Figure S6.** UV‒vis absorption spectra of time-dependent DPBF degradation under US treatment.


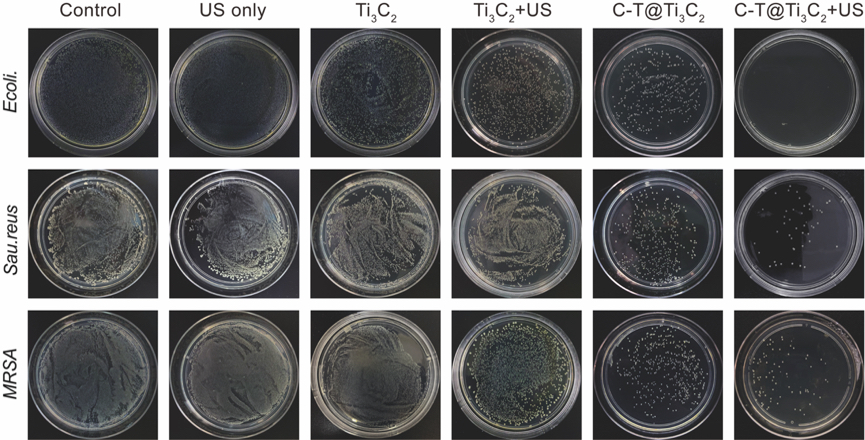


**Figure S7.** Digital photos of spread plates containing *E. coli, S. aureus,* and *MRSA* with various treatments.


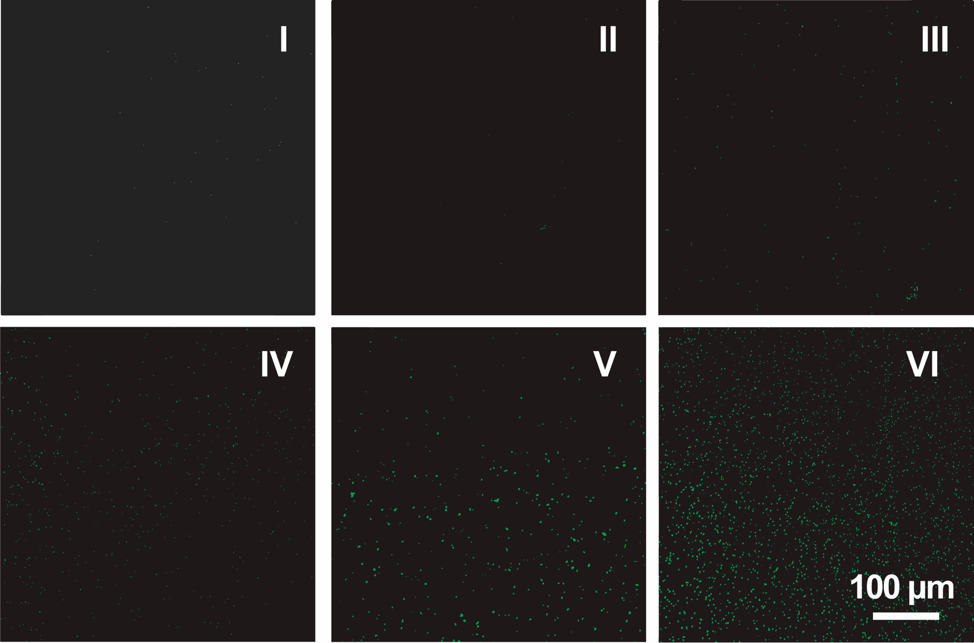


**Figure S8.** Fluorescence microscopy images of *MRSA* stained with DCFH-DA after various treatments for ROS detection (I: Control group, II: US only group, III: Ti_3_C_2_ MXene group, IV: Ti_3_C_2_ MXene + US group, V: C-T@Ti_3_C_2_ group, VI: C-T@Ti_3_C_2_ + US group).


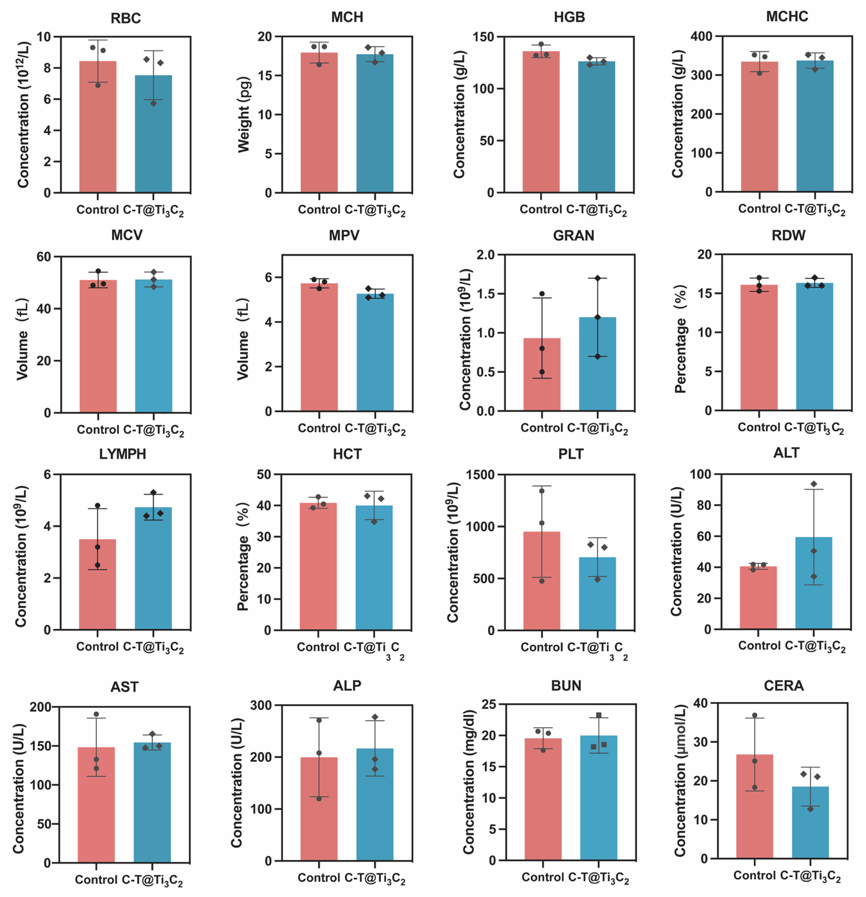


**Figure S9.** Haematological index of C57BL/6JNifdc mice intravenously administered C-T@Ti_3_C_2_ nanosheets dispersion for 14 d (n = 3). Data are presented as the mean $\text{±}$ SD. Routine blood parameters included red blood cells (RBCs), mean corpuscular haemoglobin (MCH), haemoglobin (HGB), mean corpuscular haemoglobin concentration (MCHC), mean corpuscular volume (MCV), mean platelet volume (MPV), number of neutrophils (GRAN), red cell distribution width (RDW), number of lymphocytes (LYMPH), haematocrit (HCT) and platelets (PLT). Biochemistry parameters included alanine transaminase (ALT), aspartate transaminase (AST), alkaline phosphatase (ALP), urea nitrogen (BUN), and creatinine (CREA).


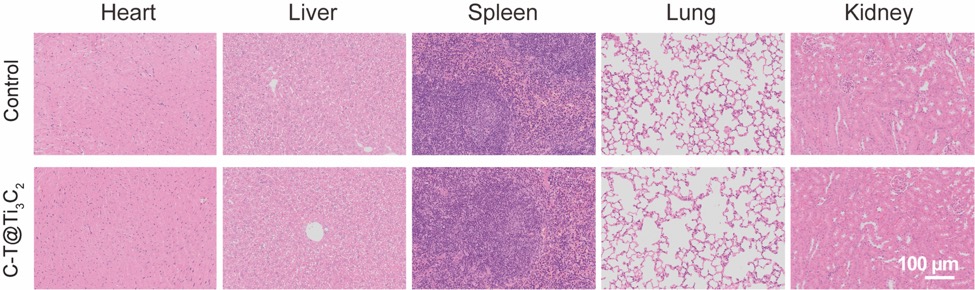


**Figure S10.** Histological assessments of the major organs (heart, liver, spleen, lung and kidney) of C57BL/6JNifdc mice after intravenous injections with C-T@Ti_3_C_2_ nanonetworks for 14 d.


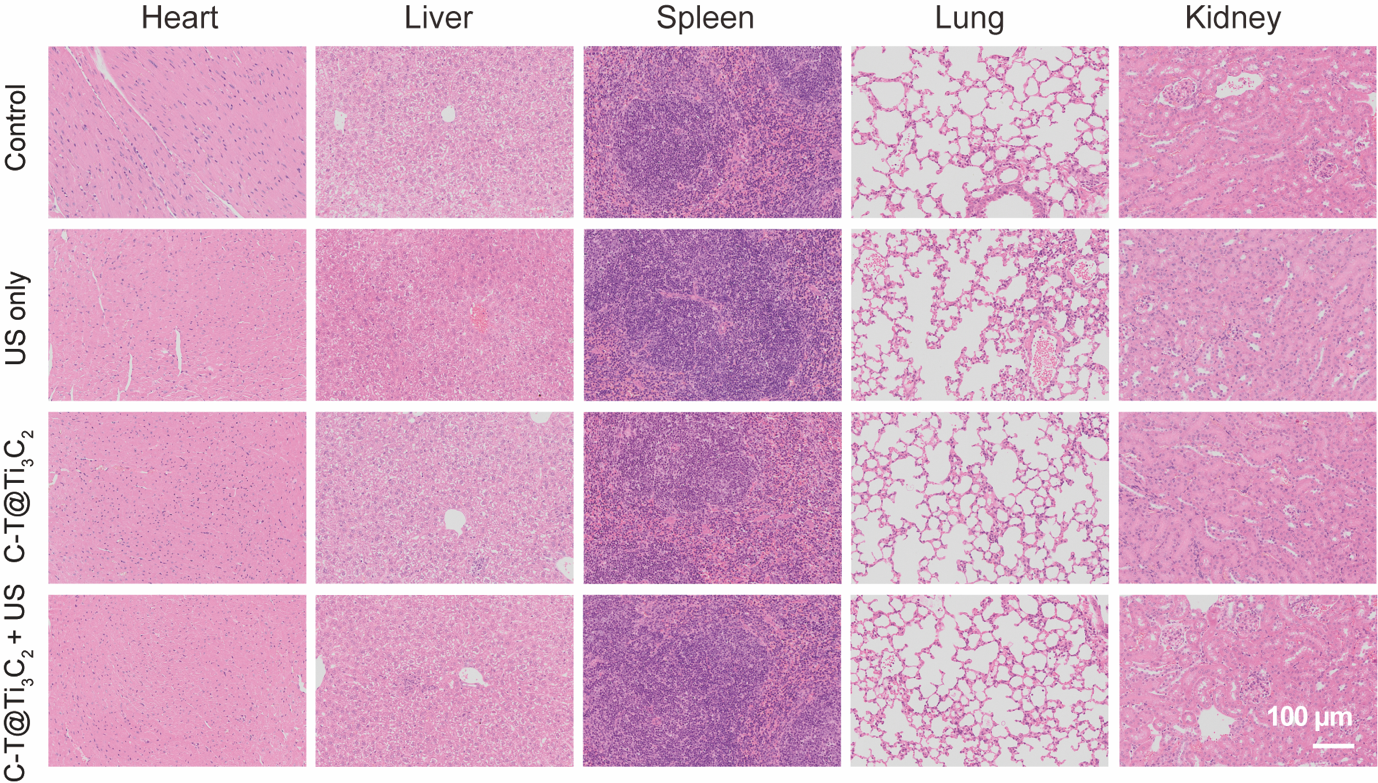


**Figure S11.** Histological assessments of the major organs (heart, liver, spleen, lung and kidney) of C57BL/6JNifdc mice after different treatments in the MRSA-infected wound model.


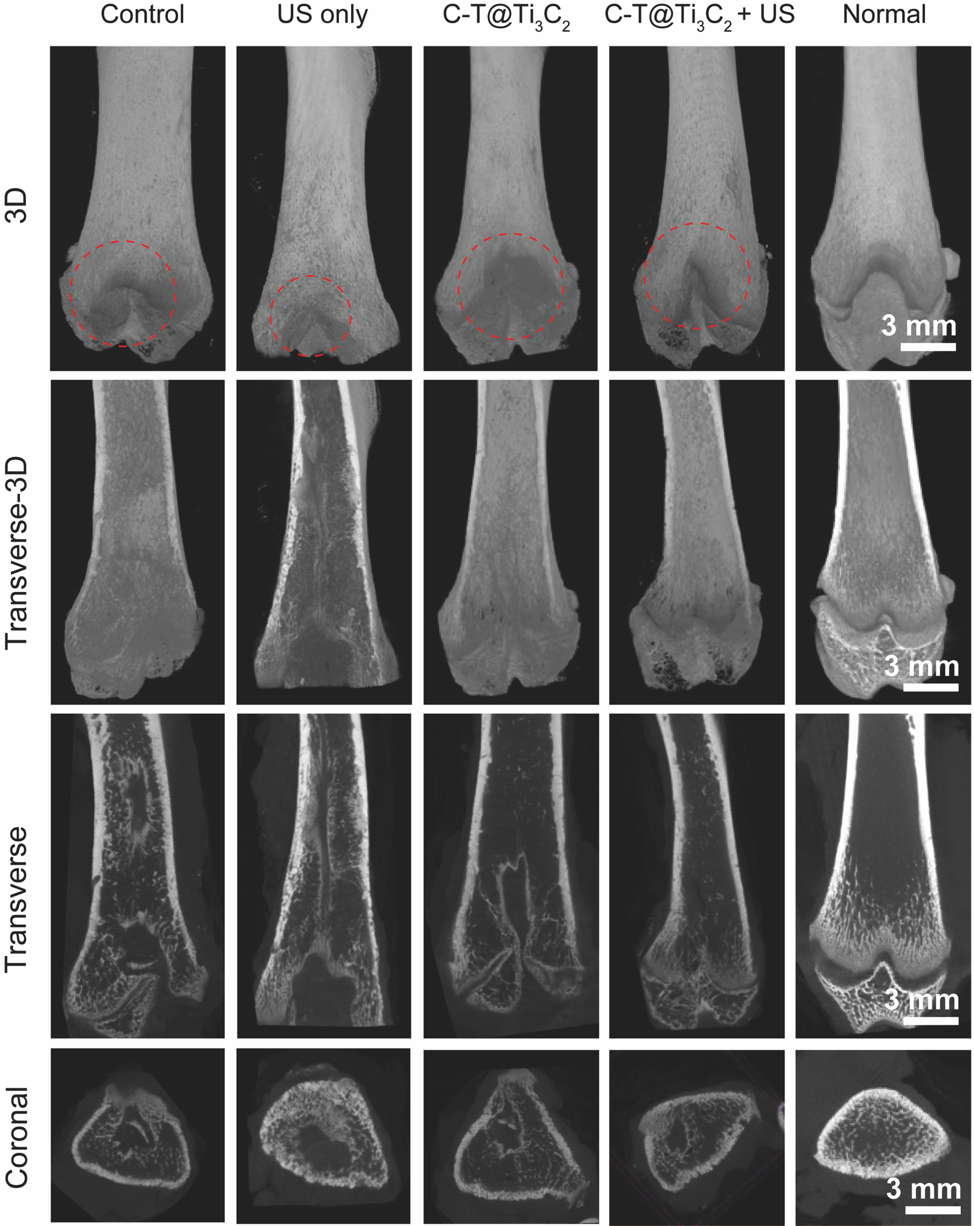


**Figure S12.** Micro-CT images of femurs in different groups after 2 weeks of treatment.


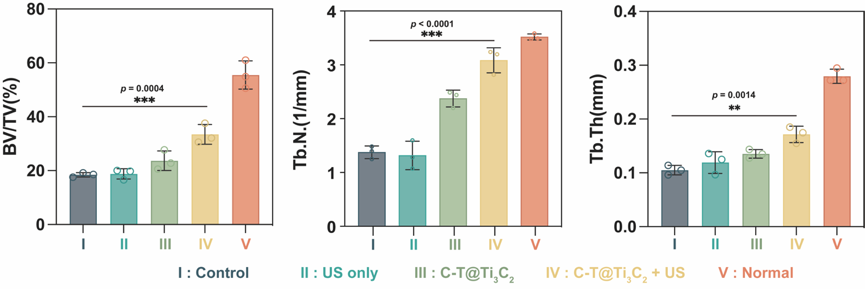


**Figure S13.** Corresponding quantitative analysis showing BV/TV, Tb. Th and Tb.N. (n=3) in different groups after surgery for 2 weeks. Data are presented as the mean $\text{±}$ SD. Statistical significance was calculated with a two-tailed t test, **P*≤ 0.05, ***P* ≤ 0.01, ****P* ≤ 0.001.

**
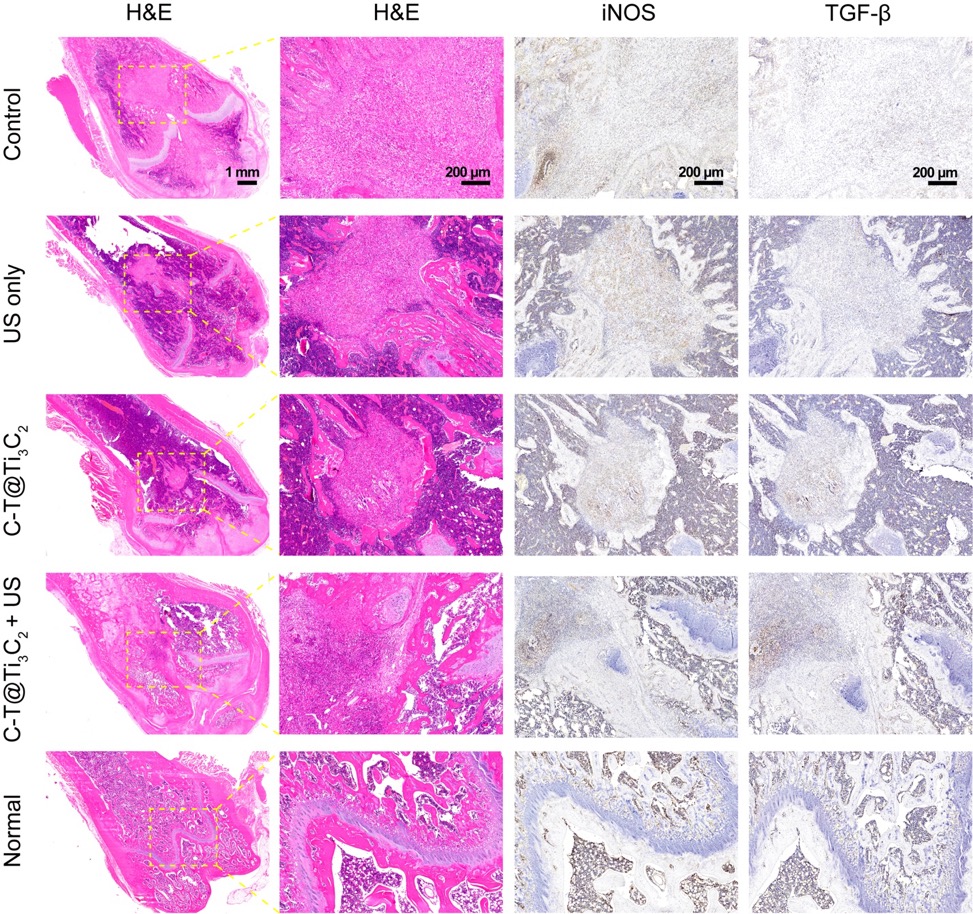
**

**Figure S14.** Histological assessments (H&E staining, iNOS and TGF-β immunohistochemical staining) of bone tissue in different groups after 2 weeks of treatment.

**
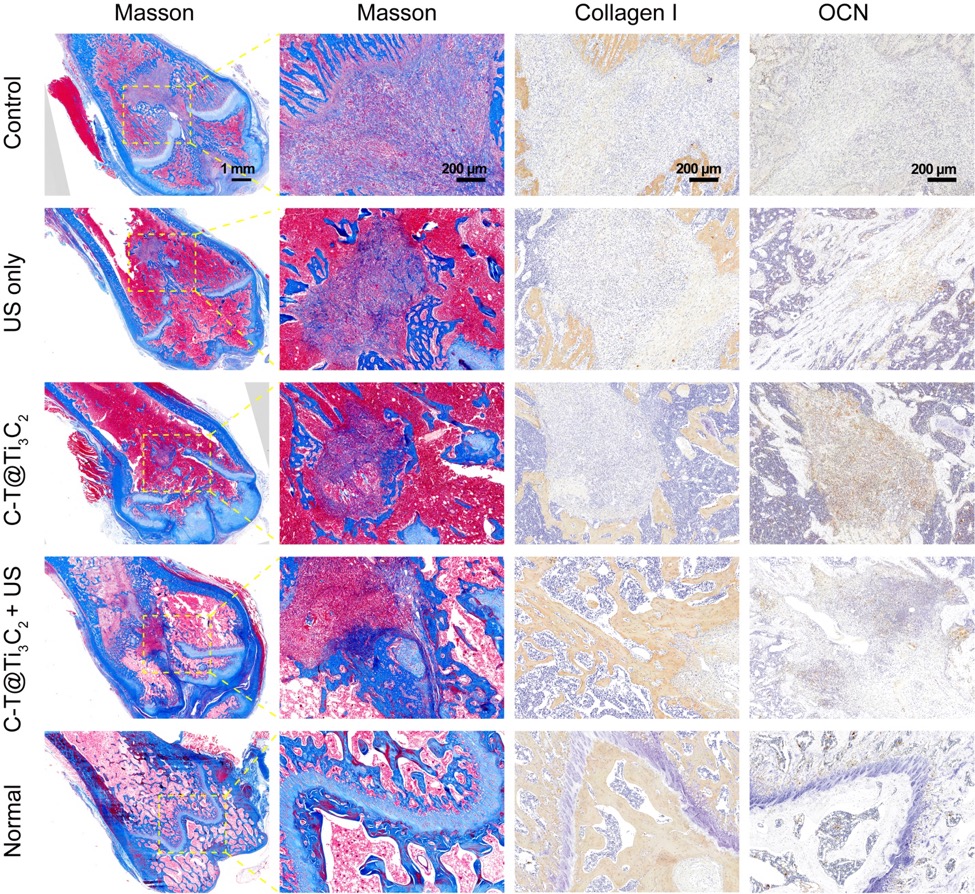
**

**Figure S15.** Histological assessments (Masson staining, collagen I and OCN immunohistochemical staining) of the bone tissue in different groups after 2 weeks of treatment.


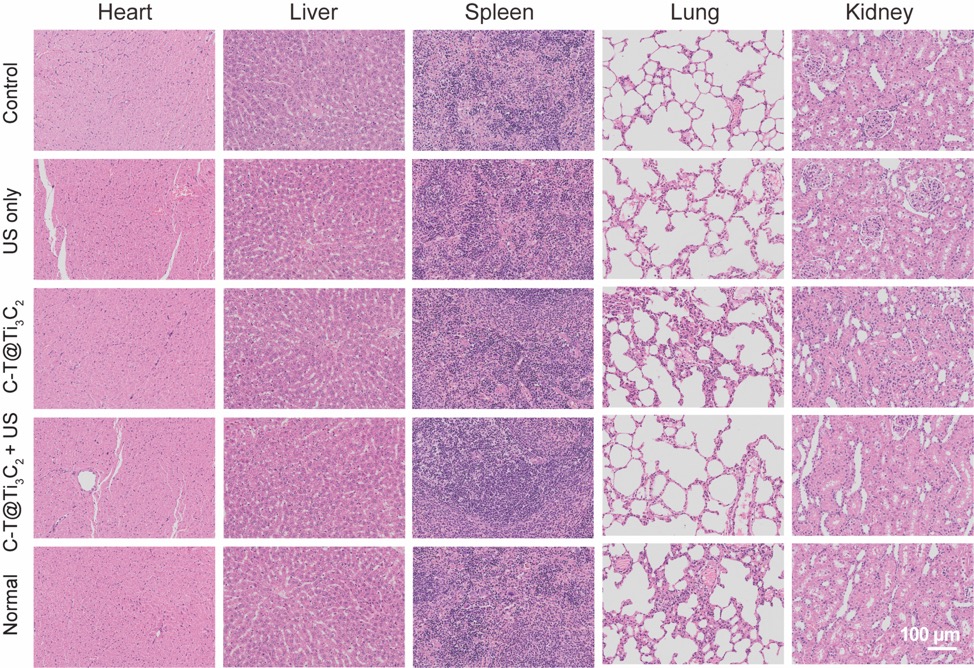


**Figure S16.** Histological assessments of the major organs (heart, liver, spleen, lung and kidney) of SD rats from the bone defect model with *MRSA* infection at 2 weeks.


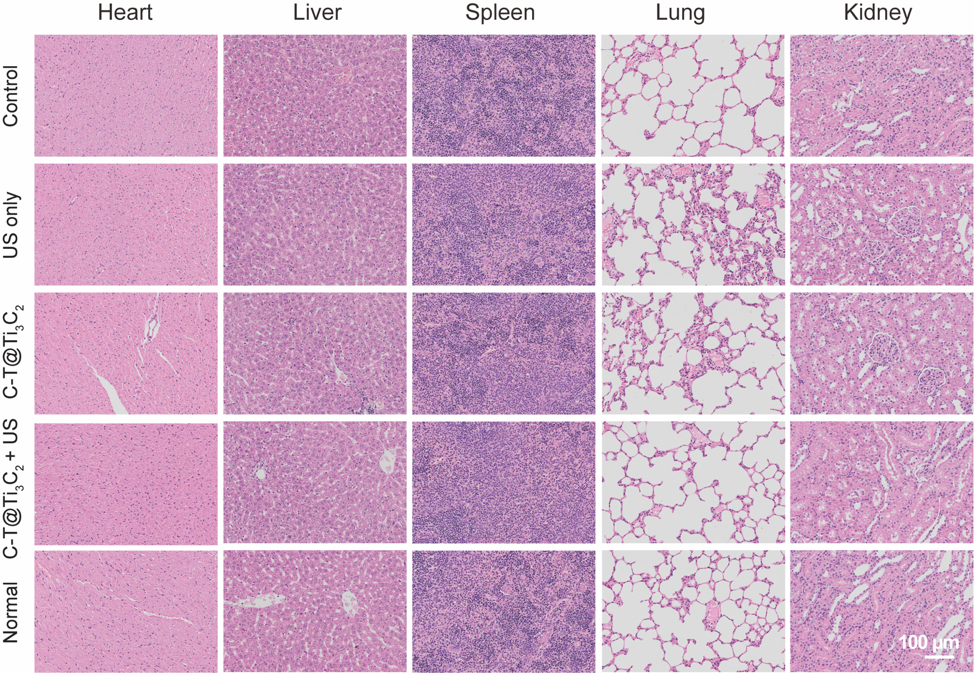


**Figure S17.** Histological assessments of the major organs (heart, liver, spleen, lung and kidney) of SD rats from the bone defect model with *MRSA* infection at 4 weeks.
